# Impact of maternal intermittent fasting during pregnancy on cardiovascular, metabolic and renal function in adult rat offspring

**DOI:** 10.1371/journal.pone.0258372

**Published:** 2022-03-10

**Authors:** Alaa Alkhalefah, Heather J. Eyre, Rezwana Hussain, Jocelyn D. Glazier, Nick Ashton

**Affiliations:** 1 Division of Cardiovascular Sciences, Faculty of Biology, Medicine and Health, Manchester Academic Health Science Centre, University of Manchester, Manchester, United Kingdom; 2 Division of Developmental Biology and Medicine, Maternal and Fetal Health Research Centre, St. Mary’s Hospital, Faculty of Biology, Medicine and Health, Manchester Academic Health Science Centre, University of Manchester, Manchester, United Kingdom; 3 Divison of Pharmacy and Optometry, Faculty of Biology, Medicine and Health, Manchester Academic Health Science Centre, University of Manchester, Manchester, United Kingdom; 4 Division of Evolution, Infection and Genomics, Faculty of Biology, Medicine and Health, Manchester Academic Health Science Centre, University of Manchester, Manchester, United Kingdom; Royal College of Surgeons in Ireland, IRELAND

## Abstract

Pregnant Muslim women are exempt from fasting during Ramadan; however a majority are reported to fast. The impact of this form of maternal intermittent fasting (IF) on fetal development and offspring health is not well defined. Using a rat model, we have shown previously that maternal IF results in fetal growth restriction accompanied by changes in placental nutrient transport function. The aim of this study was to assess cardiovascular, metabolic and renal function in adult offspring of IF-exposed dams. Food was withheld from Wistar rats from 17:00 to 09:00 daily throughout pregnancy; controls had *ad libitum* access to food. Birth weight was unaffected; however male IF pups grew more slowly up to 10 weeks of age (*P <* 0.01) whereas IF females matched their control counterparts. Systolic blood pressure (SBP), glucose tolerance and basal renal function at 14 weeks were not affected by IF exposure. When offered saline solutions (0.9–2.1%) to drink, females showed a greater salt preference than males (*P <* 0.01); however there were no differences between dietary groups. A separate group of pups was weaned onto a 4% NaCl diet. SBP increased in IF pups sooner, at 7 weeks (*P <* 0.01), than controls which became hypertensive from 10 weeks. Renal function did not appear to differ; however markers of renal injury were elevated in IF males (*P <* 0.05). Maternal IF does not affect resting cardiovascular, metabolic and renal function; but when challenged by dietary salt load male IF offspring are more prone to renal injury.

## Introduction

During the month of Ramadan, healthy adult Muslims are required to fast, abstaining from both food and drink between sunrise and sunset. Pregnant women are exempt from fasting and can elect to make up for any missed days at a later date or to pay for someone else to be fed (Fidyah) [1]. However, evidence suggests that up to 90% of women take part in the daily fast for at least part of their pregnancy [1]. The impact of maternal fasting on fetal development and the subsequent health of the child is not well defined. We have reported recently in a systematic review of the literature that maternal fasting during Ramadan is associated with a reduction in placental weight; however birth weight was not affected [2]. Other more serious but infrequent events such as stillbirth or neonatal death have not been reported, reflecting the small sample sizes of many studies, and there is a lack of longer term follow up studies looking at the health of children or adult offspring.

The availability of nutrients in the diet and the capacity of the placenta to transport resources to the developing fetus are essential for normal growth [3]. Studies on human populations subject to famine, such as that which occurred during the Dutch Hunger Winter in 1944–45, have shown that depending on the timing of exposure, fetal growth can be reduced [4]. Later in life, the adult offspring were more likely to have an increased body mass index and be at greater risk of impaired glucose tolerance, hypertension and coronary heart disease [4]. These observations have been recapitulated in a variety of animal models, including both under- and over-nutrition [5], demonstrating that the intrauterine environment is critical in determining long-term health and disease. When the supply of nutrients is inadequate, particularly during the later stages of gestation, resources are diverted to protect growth of the brain at the expense of visceral organs such as the liver and kidneys [6]. As a result, metabolism and excretory capacity are altered predisposing the offspring to diabetes and cardiovascular disease [6].

These phenomena are of interest as women are reported to participate in the Ramadan fast across each trimester of pregnancy [7–9], and differences in outcomes may be related to the gestational timing when fasting occurred [2, 8, 10]. Additionally, the timing of food consumption is altered and accompanied commonly by a change in the quantity and quality of foods consumed [7]. Hence fasting pregnant women may expose their developing babies *in utero* to an altered nutrient and metabolic environment [11, 12], which may link to the increased propensity to long-term health issues such as coronary heart disease and type 2 diabetes reported previously in the offspring of mothers who observed Ramadan fasting during pregnancy [10]. However, despite the now well-established link between maternal diet during pregnancy, the intrauterine environment and the risk of developing a range of diseases in adulthood, the potential impact of maternal fasting during Ramadan on offspring health remains poorly defined.

Hence in order to study the impact of maternal intermittent fasting (IF) on the development and subsequent health of the offspring, we have developed a rat model to mimic aspects of human IF during Ramadan [13]. Pregnant rats were subjected to IF overnight, during their active phase, for the duration of pregnancy in order to maximise the impact on the developing fetus. IF fetuses were growth restricted at gestational day (GD) 21 and placental transport efficiency (as evidenced by the fetal:placental weight ratio) was reduced. Consistent with this, placental function was affected, with changes in placental metabolites and a significantly reduced transplacental flux by the sodium-dependent system A amino acid transporter. Exposure to IF altered fetal plasma amino acid profiles, with reductions in the branched chain amino acids in particular, as well as a reduction in fetal insulin concentration. We also observed sex differences in the fetal response to maternal IF, with sex-dependent differences in placental aromatic amino acids also apparent within the IF group [13].

On the basis of these observations and the widely reported effects of maternal dietary manipulation on offspring cardiovascular [14], metabolic [15] and renal function [16] in other models of dietary-induced developmental programming, we postulated that exposure to IF during pregnancy would have an adverse effect on these physiological functions in the adult offspring. Therefore, the aim of this study was to characterise postnatal growth and to assess the impact of exposure to IF on blood pressure, glucose metabolism and renal function in the adult offspring. IF rats were also challenged with a dietary salt load in order to expose any potential dysfunction in cardio-renal regulation of blood pressure. Furthermore, as we have observed sex differences in the response of fetuses to maternal IF [13], we studied both male and female offspring to determine whether sex has any effect on the outcome.

## Materials and methods

### Ethical approval

All experiments involving animals were conducted under the authority of a project licence (PPL 40/3646) issued in accordance with the UK Animals (Scientific Procedures) Act 1986. Local ethical approval was granted by the University of Manchester Animal Welfare and Ethical Review Body. All animal work was conducted at the University of Manchester. Humane endpoints were defined in advance for each procedure and animals were monitored regularly. Care was taken to minimise suffering; no animal reached the defined humane endpoint prior to the scientific endpoint in any of the experiments.

### Animals

Virgin female Wistar rats (250–275 g, Charles River UK Ltd, Margate, Kent, UK) were acclimatised to the Biological Services Facility for one week, where they were held under a 12 h light:dark cycle (06:00–18:00) at 21–23°C and 65% humidity. All rats had free access to standard rat chow (BK001 (E) SDS Rodent Breeder and Grower, LBS Biotec, Redhill, UK: gross energy 15.10 MJ/kg, digestible energy 12.27 MJ/kg, metabolizable energy 11.24 MJ/kg) and water. Females were then paired with a Wistar male (275–300 g) until conception was confirmed by the presence of a vaginal plug (dams were checked twice daily at 09:00 and 17:00): this was designated as gestational day 1 (GD1). Females were then randomised to either intermittent fasting (IF, N = 25 rats) or control (C, N = 21 rats) groups and housed singly. Food was removed from the IF dams at 17:00 and returned at 09:00 daily, commencing at GD1 until GD22 (term is at GD23); water was available *ad libitum* throughout. Body condition scores were monitored to determine the health status of animals; the rat grimace scale and observation of normal behaviours (breathing rate, grooming, nest building, response to environmental stimuli) were used to identify distress or pain. Control animals had access to both food and water *ad libitum* throughout. 24 h food and water intake by both groups of dams was calculated by weighing food and water containers daily at the same time; consumption was calculated as the difference in weight. From GD22 onwards, dams were checked regularly throughout the day for delivery of a litter.

The overall study design including the number of dams used to generate the experimental offspring is summarised in S1 Fig.

At term following birth (on postnatal day (PD)1), litter sizes were reduced to 8 animals to minimise the potential impact of variations in litter size on milk availability and pup growth. Pups were sexed by visual estimation of the anogenital distance and randomised to be retained (4 males and 4 females where possible) or culled by stunning followed by decapitation. Organs (brain, liver and kidney) were harvested from the excess PD1 pups and weighed. All dams had free access to both food and water during the suckling period. Total litter weight was recorded daily from PD1-14 and then every other day to PD28, at which point pups were sexed as above and weaned onto standard chow. The dams were then killed by cervical dislocation under isoflurane anaesthesia (4% isoflurane in oxygen at 2 L /min). Individual offspring were weighed weekly from the age of 5 to 12 weeks, during which time blood pressure was recorded (weeks 5, 7 and 10 as described below) and the rat grimace scale and observation of normal behaviours (breathing rate, grooming, nest building, response to environmental stimuli) were used to identify distress or pain; animals were then used in one of the experimental procedures described below (S1 Fig).

### Blood pressure

Systolic blood pressure (SBP) was measured by tail cuff plethysmography (Model LE5001, PanLab, Spain) without the use of restraint, in order to minimise distress, in conscious male and female rats at 5, 7 and 10 weeks of age (control n = 80 pups from N = 11 litters; IF n = 104 pups from N = 13 litters). These rats went forward for use in one of the experiments described below, after which they were killed by cervical dislocation under anaesthesia.

### Glucose and insulin tolerance tests

Glucose (GTT) and insulin (ITT) tolerance tests were conducted in separate groups of 12 week old rats (control n = 5–6 pups per sex from N = 5–6 litters; IF n = 6–7 pups per sex from N = 6–7 litters). Rats were fasted overnight for 16 h prior to the collection of a pin-prick blood sample from the tail for the measurement of baseline glucose concentration using an Accu-Chek Mobile blood glucose monitoring system (Roche Diagnostics, West Sussex, UK). Animals then received either an i.p. injection of sterile glucose solution (10% glucose in 0.9% saline at 1 g/kg body weight for the GTT) or human insulin (0.75 unit/kg body weight, I9278, Sigma Aldrich for the ITT), following which pin-prick blood samples were collected up to 120 min post-injection. Animals experienced no more than transient discomfort as a result of blood sampling; less than 10% of total blood volume was collected. At the end of the experiment animals were killed by cervical dislocation under isoflurane anaesthesia (4% isoflurane in oxygen at 2 L /min).

### Nephron number

Nephron number was determined at PD1 (control n = 4 pups per sex from N = 4 litters; IF n = 3–5 pups from N = 5 litters) and PD12 (control n = 6 pups per sex from N = 6 litters; IF n = 5–6 pups from N = 6 litters), as described previously [17]. Pups were killed by decapitation using a method appropriate to their age (following stunning at PD1 or under isoflurane anaesthesia at PD12). Kidneys were decapsulated, minced and digested in 10 mL 1 M HCl at 37°C for 3 (PD1) or 15 min (PD12), after which 50 mL deionised water was added and homogenates were stirred gently at 4°C for 8 h. Mature glomeruli were counted in 20 x 40 μL aliquots per kidney; comma and S shaped bodies were not included.

### Renal function

Renal function was measured in 14 week old offspring (control n = 5 pups per sex from N = 5 litters; IF n = 7 pups per sex from N = 7 litters) under Inactin anaesthesia (sodium thiobutabarbital 100 mg/kg body weight i.p., T133, Sigma Aldrich), as described previously [17]. A surgical plane of anaesthesia was confirmed through the absence of a pedal reflex; this was checked at regular intervals throughout the experiment. A priming dose of clearance markers (0.148 MBq ^3^H inulin, PerkinElmer, Monza, Italy and 12 mg para-aminohippuric acid (PAH), A3759, Sigma Aldrich in 0.2 mL 0.9% saline) was administered intravenously following which animals were infused continuously with 0.9% saline containing ^3^H inulin (0.0333 MBq/mL) and PAH (1 mg/mL) at 50 μL/min for a 3 h equilibration period. Thereafter urine samples were collected via a bladder catheter at 15 min intervals over 3 h; arterial blood samples (400 μL) were collected once every hour over 3 h. Blood pressure was recorded continuously via a carotid artery catheter (Powerlab 800/s, ADInstruments, Hastings, East Sussex, UK). Haematocrit was measured at the end of the experiment for the subsequent calculation of effective renal blood flow. Urine and plasma samples were analysed for ^3^H inulin activity (2000CA Tri-Carb Liquid Scintillation Analyser, Canberra Industries, Meriden, CT, USA), PAH (standard colorimetric assay), Na^+^ and K^+^ concentrations (flame photometer model 420, Sherwood Scientific Ltd, Cambridge, UK) and osmolality (Vapour pressure osmometer model 5500, Wescor, Inc, Logan, UT, USA); plasma was also analysed for protein concentration (absorbance at 280 nm, Nanodrop 2000c spectrophotometer, Thermo Fisher Scientific, Waltham, MA, USA). Animals were killed by cervical dislocation at the end of the experiment.

### Salt preference and aversion threshold

A two-bottle choice protocol was used to determine salt preference and total fluid intake, as described previously [18]. At 7 weeks of age rats (control n = 5 pups per sex from N = 5 litters; IF n = 6 pups per sex from N = 6 litters) were housed individually and offered bottles containing sterile water or 0.9% saline. Following 2 days acclimatisation, daily fluid intake was recorded over 5 consecutive days. The concentration threshold for salt aversion was subsequently determined by offering the rats a choice between sterile water and increasing concentrations of saline (from 0.9% to 2.1% in 0.3% increments every 3 days over 12 days) to determine the concentration at which they switched their preference from saline to water. Body condition scores and skin turgor were monitored to determine the hydration status of animals; the rat grimace scale and observation of normal behaviours (breathing rate, grooming, nest building, response to environmental stimuli) were used to identify distress or pain. At the end of the experiment animals were killed by cervical dislocation under isoflurane anaesthesia (4% isoflurane in oxygen at 2 L /min).

### Extracellular fluid volume

Extracellular fluid volume was determined in 12 week old offspring (control n = 5 pups per sex from N = 5 litters; IF n = 6 pups per sex from N = 6 litters) under Inactin anaesthesia (100 mg/kg body weight, i.p.) as described previously [18]. Following a laparotomy, both sets of renal vessels were occluded using 3–0 mersilk and the abdomen was closed. 0.222 MBq ^3^H inulin in 350 μL 0.9% saline was injected intravenously, followed by a saline flush (total volume of injectate was 500 μL). Following a 90 min equilibration period, blood samples (50 μL) were collected every 10 min over 60 min to measure plasma ^3^H inulin activity and to calculate the dilution of the injected ^3^H inulin. Animals were killed by cervical dislocation at the end of the experiment and a sample of urine was taken from the bladder to confirm occlusion of the renal vessels.

### High salt diet

The impact of dietary salt loading on SBP and renal function was determined in a separate group of rats. These animals were either exposed to maternal IF *in utero* or a control *ad libitum* diet as described above. Following weaning at 4 weeks, rats were randomised to receive either a high salt (4% NaCl) diet (BK001 (E) 4% NaCl SDS Rodent Breeder and Grower, LBS Biotec, Redhill, UK) or a standard (1% NaCl) rat chow diet (BK001 (E) SDS Rodent Breeder and Grower, LBS Biotec, Redhill, UK) until 14 weeks of age (control n = 5 pups per sex per diet from N = 5 litters; IF n = 6 pups per sex per diet from N = 6 litters). Body condition scores and skin turgor were monitored to determine the hydration status of animals; the rat grimace scale and observation of normal behaviours (breathing rate, grooming, nest building, response to environmental stimuli) were used to identify distress or pain. SBP was measured as described above at 5, 7 and 10 weeks of age. At 12 weeks of age the rats were housed individually in metabolism cages until they had voided sufficient urine for analysis (3 mL typically collected over 3 h in order to minimise distress; no animal was held in a metabolism cage for more than 4 h). Renal function was then determined in anaesthetised rats at 14 weeks of age, as described above. Animals were killed by cervical dislocation at the end of the experiment.

### Urine analysis

Urinary creatinine concentration was determined using a colorimetric assay, according to the manufacturer’s instructions (DetectX urinary creatinine kit, Arbor Assays, MI, USA). Urinary albumin concentration was determined using a rat albumin ELISA kit, according to the manufacturer’s instructions (Bethyl Laboratories, Inc, TX, USA). Urinary neutrophil gelatinase-associated lipocalin (NGAL) concentration was determined using a rat Lcn2 ELISA kit, according to the manufacturer’s instructions (RAB0906, Sigma Aldrich, UK).

### Statistical analysis

Data are presented as box (with median) and whisker plots (whiskers represent 5^th^ and 95^th^ centiles) or as mean ± SEM. N represents the dam or litter and n represents the offspring from a litter. Where measurements were recorded for the whole litter (e.g. body weight) the litter average is presented. In all other experiments data are representative of individual offspring. No more than 2 rats of each sex from any given litter were included in an experimental group. Distribution of the data was evaluated using a Shapiro-Wilk test, after which two-way ANOVA (with repeated measures where appropriate) and Tukey tests or Kruskal-Wallis and Dunn’s multiple comparison tests were applied, as appropriate, where more than 2 groups were compared. For comparisons between 2 groups Student’s unpaired t-tests were used. Data were analysed using SPSS (version 22.0, IBM SPSS Statistics, IBM United Kingdom Ltd, Hampshire, UK) and GraphPad Prism (version 7.0, GraphPad Software, Inc, La Jolla, CA, USA); statistical significance was taken as *P <* 0.05.

## Results

### Maternal intermittent fasting impairs maternal weight gain and slows growth of male offspring

Pregnant rats subjected to IF ate 25 ± 1% less food over the course of gestation than controls with *ad libitum* access to food (*P* < 0.001, S2A Fig). Their *ad libitum* water intake tended to be lower too, reaching statistical significance (*P* < 0.05) over several days in the second half of gestation (S2B Fig). As a result, IF dams gained significantly less weight than controls from GD18 onwards (*P* < 0.001, S2C Fig). Despite this reduction in weight gain by IF dams, their litter sizes and the body weight of newborn pups did not differ significantly from that of control dams (Table 1), even though all IF dams delivered ~0.5 day earlier than controls. Although body weight did not differ between IF and control pups at PD1, organ growth, particularly that of the brain, was impaired. The brain:liver weight ratios of both male (*P* < 0.05) and female (*P* < 0.05) IF rats were significantly lower than that of the controls (Table 2). This reflected a significant reduction in brain weight (*P* < 0.05) rather than liver weight in females (*P* > 0.05). Males exhibited a similar pattern; however the reduction in brain weight did not reach statistical significance. Kidney weight was also reduced significantly in both male (*P* < 0.01) and female (*P* < 0.001) IF rats compared with controls (Table 2).

**Table 1:** Litter size and pup birth weight at PD1 in control and IF offspring.

	Control (N = 11)	IF (N = 13)
Litter size	15 ± 1	13 ± 1
Pup body weight (g)	6.5 ± 0.2	6.3 ± 0.2

Data are shown as mean ± SEM. Pup body weight represents the average weight per litter; N is the number of litters. Statistical comparisons were by unpaired t-test. No significant differences were identified.

**Table 2:** Organ weights at PD1 in control and IF offspring.

	Control (N = 9)		IF (N = 11)	
	Male	Female	Male	Female
Kidney (mg/g body weight)	11.3 ± 0.3	11.6 ± 0.3	9.6 ± 0.3**	9.7 ± 0.3***
Brain (mg/g body weight)	41.3 ± 1.6	44.6 ± 1.3	39.7 ± 0.8	40.8 ± 0.6*
Liver (mg/g body weight)	36.7 ± 0.7	37.9 ± 0.6	39.1 ± 1.0	38.9 ± 1.4
Brain/Liver ratio	1.13 ± 0.06	1.18 ± 0.04	1.02 ± 0.04*	1.05 ± 0.04*

Data are shown as mean ± SEM. Values represent the average per litter; N is the number of litters. Statistical comparisons were by two-way ANOVA and Tukey’s test.

* *P* < 0.05

** *P* < 0.01

*** *P* < 0.001 IF vs control.

**Fig 1:**
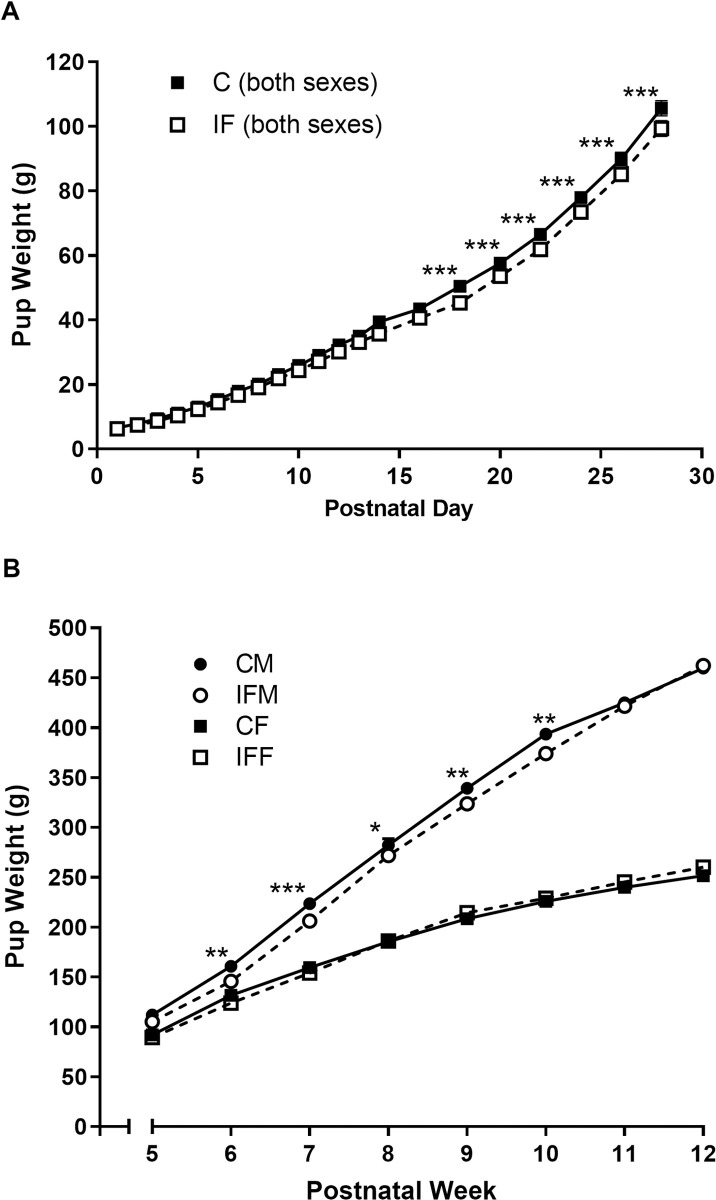
Body weight prior to (A) and after weaning (B) up to 12 weeks of age in the offspring of control and IF pregnancies. (A) Body weight is shown as the litter mean up to weaning at 4 weeks of age (control N = 11 closed square, IF N = 13 open square). (B) Pups were then split by sex (male circle, female square) and weight was recorded from 5 to 12 weeks of age (control N = 10 closed symbols, IF N = 13 open symbols). Data are presented as mean ± SEM, except when SEM falls within the size of the symbol. Statistical comparisons were by two-way ANOVA with repeated measures and Tukey’s test. * *P* < 0.05, ** *P* < 0.01, *** *P* < 0.001 IF vs control.

Up to 16 days of age, the growth curves of control and IF pups were similar (Fig 1A). However, from PD18 onwards IF pups gained significantly less weight than controls (*P* < 0.001). Pups were divided into males and females when weaned at 4 weeks old. At this point it became apparent that the lower rate of weight gain was driven by the males, since female IF pups gained weight at the same rate as their control counterparts from weeks 5 to 12 whereas IF males were significantly lighter than control males (*P* < 0.05, Fig 1B).

### Maternal intermittent fasting does not affect offspring blood pressure or insulin resistance

**Fig 2:**
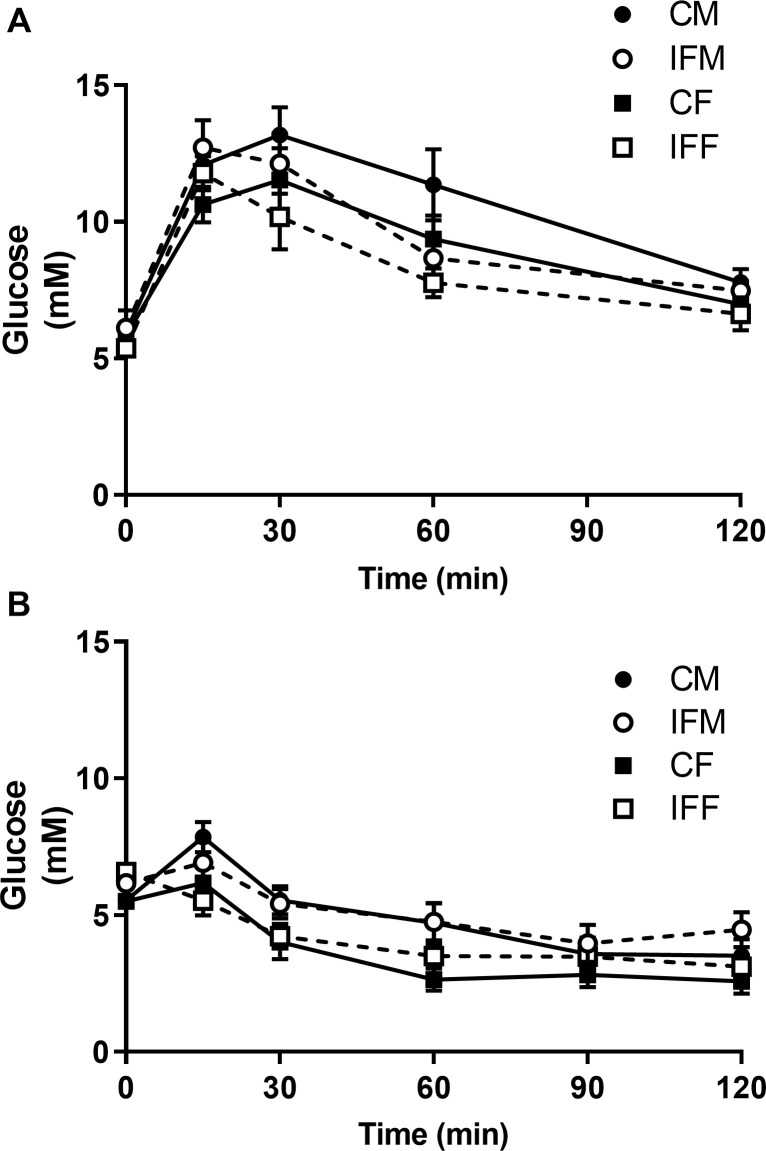
Blood glucose concentration over 2 h following i.p. injection of glucose (A) or insulin (B) in control and IF offspring at 12 weeks of age. Following a 16 h overnight fast male (circle) and female (square) rats were injected with either glucose (1 g/kg body weight, control n = 6 closed symbols, IF n = 7 open symbols) or insulin (0.75 unit/kg body weight, control n = 5, IF n = 6). Blood samples were collected over 120 min. Data are presented as mean ± SEM. Area under the curve was calculated and compared by two-way ANOVA (glucose) or Kruskal-Wallis test (insulin). No significant differences were identified.

Neither SBP nor heart rate differed between control and IF offspring at 5, 7 and 10 weeks of age (Table 3). Blood pressure tended to increase with age, but there were no differences between the dietary groups or between sexes. Glucose (Fig 2A) and insulin (Fig 2B) tolerance tests did not reveal any differences between the dietary groups or between sexes either. The area under the blood glucose concentration curve of male and female IF offspring, following injection of either glucose or insulin, did not differ significantly from that of their control counterparts.

**Table 3:** Systolic blood pressure (SBP) and heart rate in control and IF offspring at 5, 7 and 10 weeks of age.

	Control (N = 5)		IF (N = 6)	
	Male	Female	Male	Female
SBP (mmHg)				
Week 5	104 ± 2	102 ± 2	101 ± 2	103 ± 2
Week 7	112 ± 1	109 ± 1	110 ± 1	109 ± 1
Week 10	109 ± 2	108 ± 1	109 ± 1	107 ± 1
Heart Rate (bpm)				
Week 5	431 ± 8	430 ± 6	435 ± 10	433 ± 6
Week 7	440 ± 3	434 ± 4	439 ± 2	434 ± 4
Week 10	432 ± 3	429 ± 6	438 ± 2	434 ± 1

Data are shown as mean ± SEM. Values represent the average per litter; N is the number of litters. Statistical comparisons were by two-way ANOVA (SBP) or Kruskal-Wallis test (heart rate). No significant differences were identified.

### Nephron number and basal renal function are not altered in IF offspring

Nephron number increased with age (*P* < 0.001); but it did not differ between control and IF offspring either at birth (PD1), when the kidney is still undergoing rapid nephrogenesis, or when nephrogenesis was complete at PD12 (Table 4) [19].

**Table 4:** Nephron number in control and IF offspring at PD1 and PD12.

	Control		IF	
	Male	Female	Male	Female
PD1	2625 ± 217	2955 ± 219	2513 ± 117	2450 ± 125
	N = 4	N = 4	N = 5	N = 5
PD12	22938 ± 497	22488 ± 499	22288 ± 570	22200 ± 584
	N = 6	N = 6	N = 6	N = 6

Data are shown as mean ± SEM number of glomeruli per kidney. Values represent the average per litter; N is the number of litters. Statistical comparisons were by three-way ANOVA. Nephron number increased significantly with age (P < 0.001); however no significant differences were identified within each age group.

Renal clearance was used to assess baseline renal function in anaesthetised male and female IF offspring at 14 weeks of age. In agreement with measurements made in conscious animals at 5–10 weeks of age (Table 3), mean arterial blood pressure recorded directly under anaesthesia did not differ between IF and control rats of either sex (Table 5). Plasma sodium and protein concentrations, and plasma osmolality did not differ significantly between groups (Table 5). However, the plasma potassium concentration of male IF offspring was significantly higher than that of control males (*P* < 0.05, Table 5). Haematocrit was significantly lower (*P* < 0.05, Table 5) in female rats compared with their male counterparts.

**Table 5:** Mean arterial pressure (MAP), plasma electrolyte concentrations, osmolality, protein concentration and haematocrit in anaesthetised control and IF offspring during renal clearance measurements at 14 weeks of age.

	Control (N = 5)		IF (N = 7)	
	Male	Female	Male	Female
MAP (mmHg)	125 ± 4	121 ± 3	119 ± 5	129 ± 3
Na^+^ (mmol/L)	141 ± 3	134 ± 4	144 ± 3	141 ± 3
K^+^ (mmol/L)	3.0 ± 0.2	3.1 ± 0.2	3.7 ± 0.1*	3.0 ± 0.1^#^
Osmolality (mOsm/kg H_2_O)	357 ± 11	357 ± 9	363 ± 8	361 ± 6
Protein (g/100 mL)	4.5 ± 0.1	4.1 ± 0.1	4.3 ± 0.2	3.9 ± 0.2
Haematocrit (%)	46.8 ± 0.7	41.9 ± 1.0^#^	50.1 ± 1.0	43.1 ± 1.2^#^^#^^#^

Data are shown as mean ± SEM. Values represent the average per litter; N is the number of litters. Statistical comparisons were by two-way ANOVA and Tukey’s test or Kruskal-Wallis and Dunn’s multiple comparison test.

* *P* < 0.05 IF vs control

^#^ *P* < 0.05

^###^ *P* < 0.001 female vs male.

**Fig 3:**
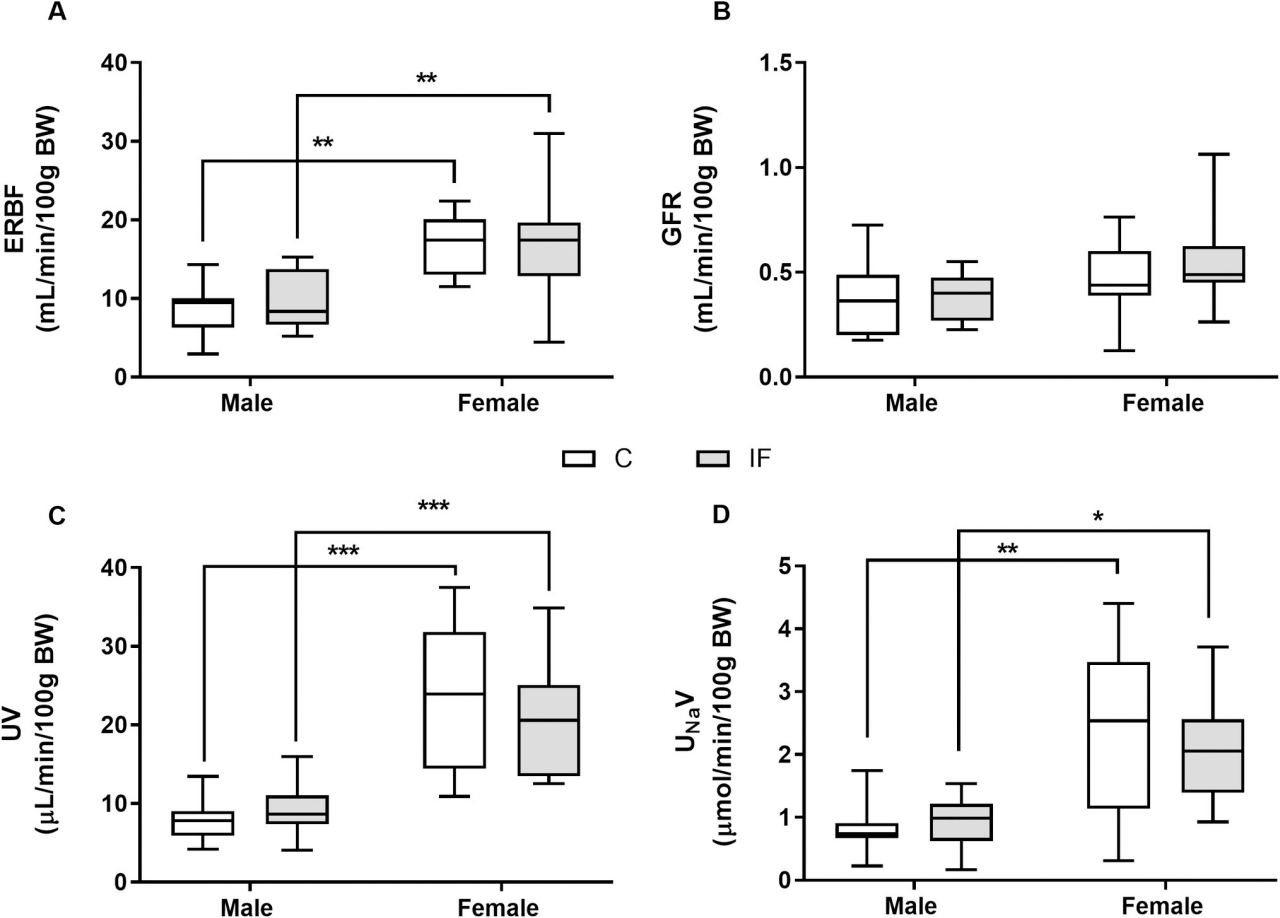
Effective renal blood flow (A), glomerular filtration rate (B), urine flow rate (C) and sodium excretion rate (D) in anaesthetised control and IF offspring at 14 weeks of age. Renal haemodynamics (A-B) and urinary excretion rates (C-D) were measured over 3 h during continuous infusion of 0.9% saline at 50 μL/min in male and female control (N = 5 open boxes) and IF (N = 7 shaded boxes) offspring. Statistical comparisons were by two-way ANOVA and Tukey’s test or Kruskal-Wallis and Dunn’s multiple comparison test (sodium excretion). * *P* < 0.05, ** *P* < 0.01, *** *P* < 0.001 male vs female.

The measured renal variables were stable over the 3 h experimental period, therefore for clarity data are shown as the average over time. Effective renal blood flow (ERBF, Fig 3A) was significantly higher in females compared with males (*P* < 0.01), but did not differ between IF and controls. In contrast there were no significant differences in glomerular filtration rate (GFR, Fig 3B) between the sexes, or dietary groups. Both urine flow rate (UV, Fig 3C) and urinary sodium excretion rate (U_Na_V, Fig 3D) were significantly greater in females compared with males (*P* < 0.05), as were the potassium and osmolar excretion rates (*P* < 0.01, S3A and S3C Fig), but there were no differences between IF and control offspring. Fractional excretion of sodium was significantly higher in control females compared with control males (control male N = 5, 2.1 ± 0.4 vs control female N = 5, 4.2 ± 0.7%, *P* < 0.05), which was not reflected by the IF animals (IF male N = 7, 1.7 ± 0.3 vs IF female N = 7, 2.8 ± 0.3%, *P* > 0.05), but there were no differences in fractional excretion of potassium or free water clearance between the sexes or dietary groups (S3B and S3D Fig).

### Females have a greater preference for salt

**Fig 4:**
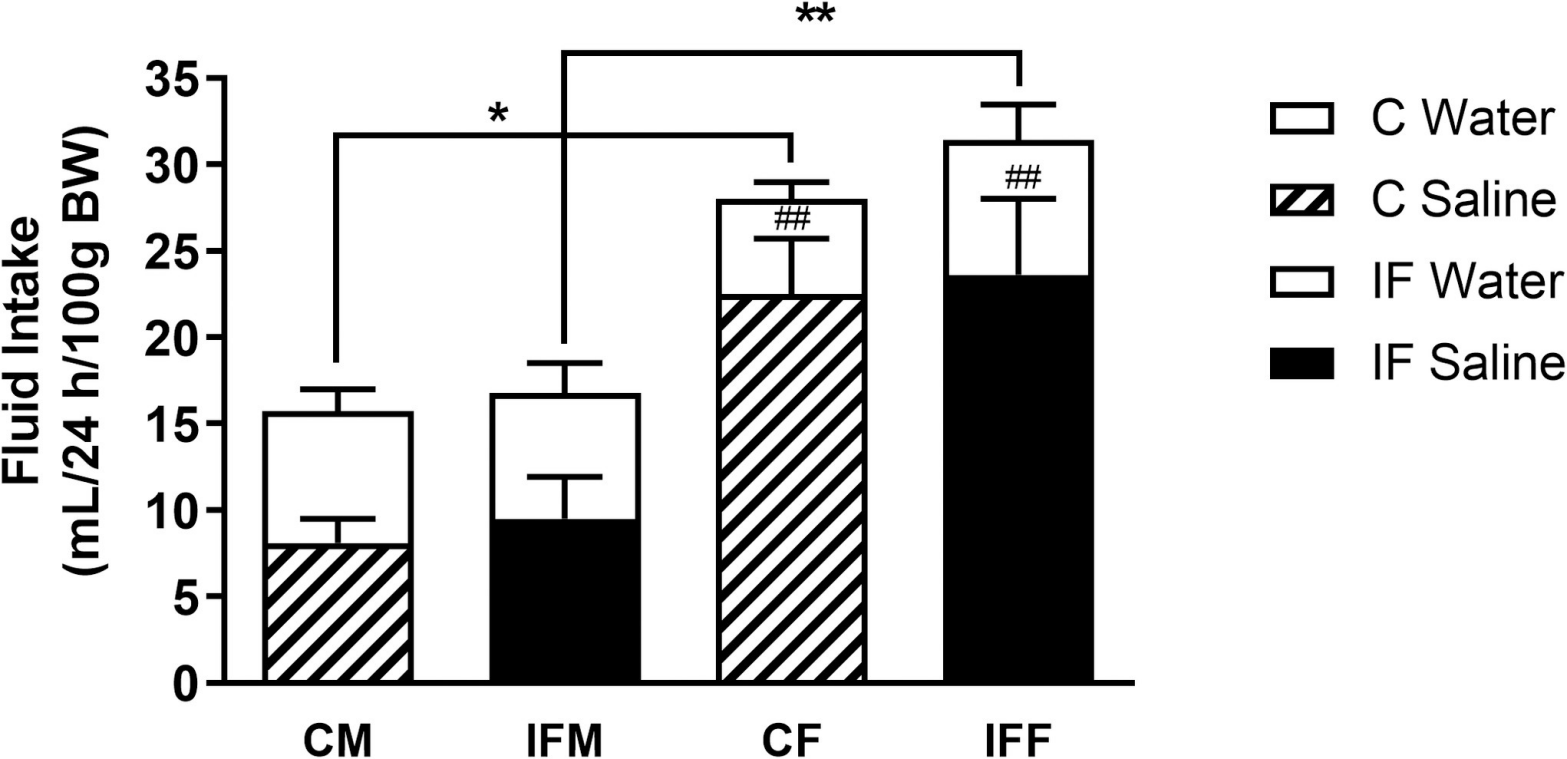
Salt preference and fluid intake in control and IF offspring at 7 weeks of age. Male (M) and female (F) rats were offered a choice of water or 0.9% saline as drinking fluid (control N = 5, open bar = water, hatched bar = saline intake; IF N = 6, open bar = water, solid bar = saline intake). Data are shown as the mean + SEM of 5 consecutive days. Statistical comparisons were by two-way ANOVA and Tukey’s test. * *P* < 0.05, ** *P* < 0.01 total fluid intake by male vs female; ^##^ *P* < 0.01 saline vs water intake.

Salt preference and the salt aversion threshold were assessed using two-bottle choice tests. When given the opportunity to choose between 0.9% saline and water to drink, female rats not only drank more fluid per 100 g body weight than males (*P* < 0.05), they also showed a stronger preference for saline over water (*P* < 0.01, Fig 4). Females of both groups drank 78–87% more fluid in total, relative to body weight, than their male counterparts. The amount of saline that they drank, as a proportion of the total fluid intake, was 33–54% greater than that ingested by males. Thus, females of both groups showed a stronger preference for 0.9% saline than males.

Interestingly, the greater preference for 0.9% saline shown by control females was not reflected by a higher threshold for salt aversion. When saline of increasing % was offered to rats, the threshold at which they switched their preference from saline to water was between 1.5% and 1.8% for control and IF males, as well as IF females (S4 Fig). However, in control females, the aversion threshold was significantly lower (*P* < 0.01), falling between 1.2% and 1.5% (S4C Fig).

Despite showing a greater preference for salt, the extracellular fluid volumes of female control (N = 4, 21.5 ± 1.0) and female IF rats (N = 6, 23.5 ± 1.3) did not differ from their respective male counterparts (control male N = 4, 24.6 ± 1.4; IF male N = 7, 21.8 ± 1.0 mL/100 g body weight), nor were there significant differences between the dietary groups.

### Salt loading has sex-dependent effects on body weight and blood pressure

**Fig 5:**
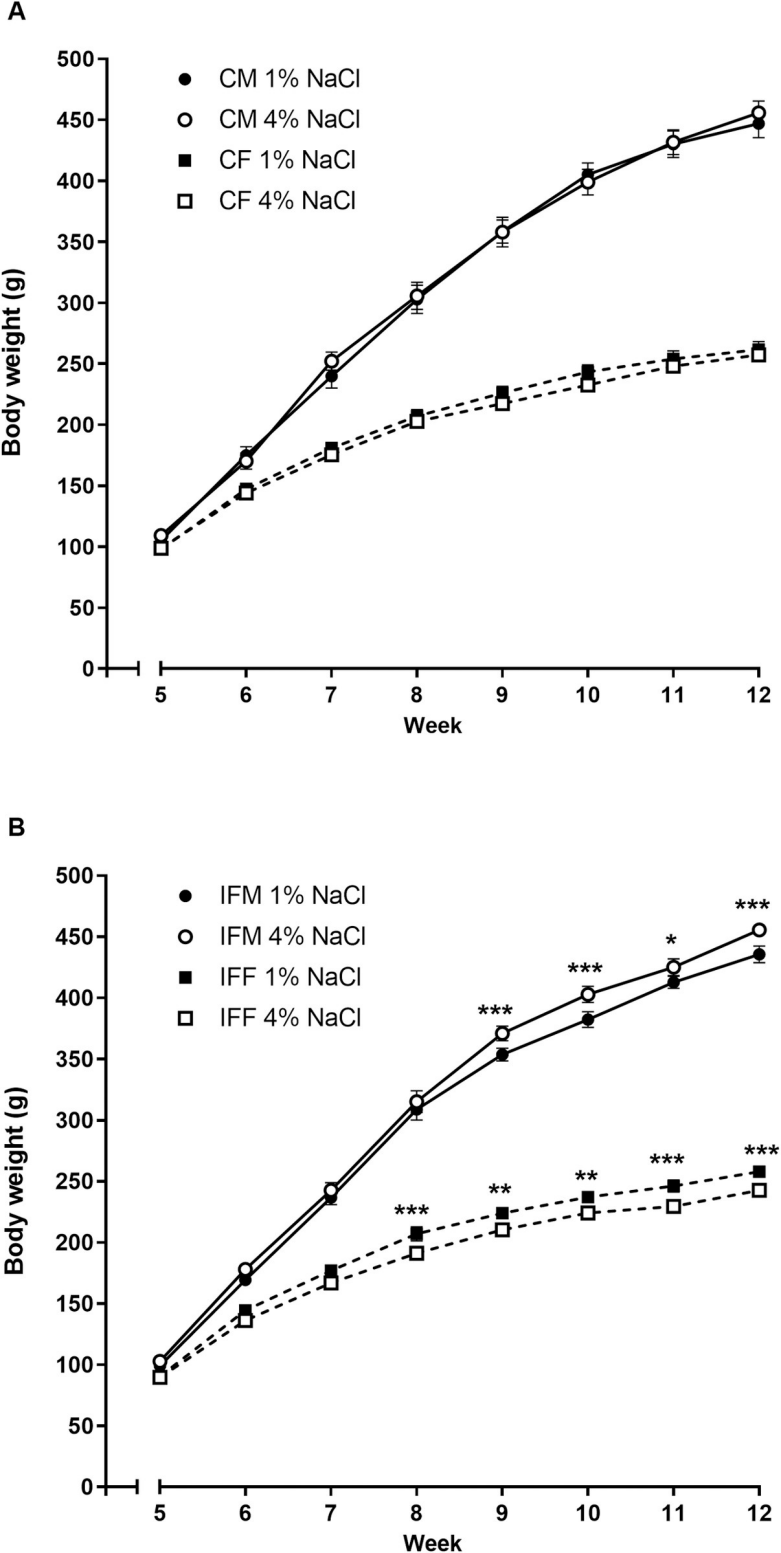
Body weight of control (A) and IF (B) offspring fed either a 1% or 4% salt diet from 4 weeks to 12 weeks of age. Male (circles) and female (squares) control (N = 5 per diet) and IF (N = 6 per diet) rats were weaned at 4 weeks of age onto a diet containing either 1% NaCl (NS–normal salt, solid symbols) or 4% NaCl (HS–high salt, open symbols). Data are presented as mean ± SEM, except when SEM falls within the size of the symbol. Statistical comparisons were by two-way ANOVA with repeated measures and Tukey’s test. * *P* < 0.05, ** *P* < 0.01, *** *P* < 0.001 HS diet vs NS diet.

In order to challenge the cardio-renal systems of IF rats, and thus identify any underlying impairment, rats were weaned at 4 weeks of age onto a high salt diet containing 4% NaCl or a control standard diet containing 1% NaCl. There were some fluctuations in food intake, but overall, all rats receiving a high salt diet ate similar quantities of food to their counterparts on a standard salt diet, when adjusted for body weight (S5A and S5B Fig). In contrast, rats eating a high salt diet drank significantly more water, per 100 g body weight, than their counterparts on a standard salt diet (S5C and S5D Fig). However, there were no differences between the sexes or IF and control rats eating the high salt diet. Control male rats were heavier and gained more weight over the 7 week experimental period than control females; however dietary salt intake did not affect the body weight of either sex (Fig 5A). In contrast, dietary salt had a sex-dependent effect on body weight in IF rats. Males were heavier than females throughout, as expected; however while IF males on a high salt diet gained more weight than their counterparts on a standard salt diet (*P* < 0.01), IF females on a high salt diet gained less weight than their counterparts on a standard salt diet (*P* < 0.01, Fig 5B).

After one week on the high salt diet (5 weeks of age), SBP did not differ between those rats fed 4% NaCl (Table 6) and those on the standard diet (Table 3). Subsequently, IF rats responded more robustly to the high salt diet than controls rats, with both sexes of IF offspring exhibiting significantly higher (*P* < 0.01) SBP than their counterparts on a standard salt diet at 7 weeks of age (Table 3 vs Table 6). SBP did not differ between control males on a high salt versus standard salt diet at 7 weeks of age, and control females fed a high salt diet only showed a modest, albeit significant increase in SBP (*P* < 0.05, Table 3 vs Table 6). It was not until 10 weeks of age that all rats fed a high salt diet exhibited significantly higher SBP (*P* < 0.01) than their counterparts on a standard salt diet (Table 3 vs Table 6). Despite the difference in timescales for the onset of hypertension when rats were fed a high salt diet, overall there were no statistically significant differences between IF and control rats of either sex. Heart rate was similarly unaffected (Table 6).

**Table 6:** Systolic blood pressure (SBP) and heart rate in control and IF offspring fed a 4% NaCl diet at 5, 7 and 10 weeks of age.

	Control (N = 5)		IF (N = 6)	
	Male	Female	Male	Female
SBP (mmHg)				
Week 5	108 ± 2	107 ± 2	106 ± 3	107 ± 3
Week 7	115 ± 2	114 ± 2	117 ± 2	116 ± 2
Week 10	120 ± 3	118 ± 2	123 ± 2	118 ± 3
Heart Rate (bpm)				
Week 5	437 ± 4	435 ± 3	443 ± 6	433 ± 4
Week 7	437 ± 2	433 ± 3	441 ± 2	432 ± 6
Week 10	436 ± 1	432 ± 4	441 ± 2	436 ± 5

Data are shown as mean ± SEM. Values represent the average per litter; N is the number of litters. Statistical comparisons were by two-way ANOVA. No significant differences were identified.

### Salt loading causes renal injury in male IF offspring

**Fig 6:**
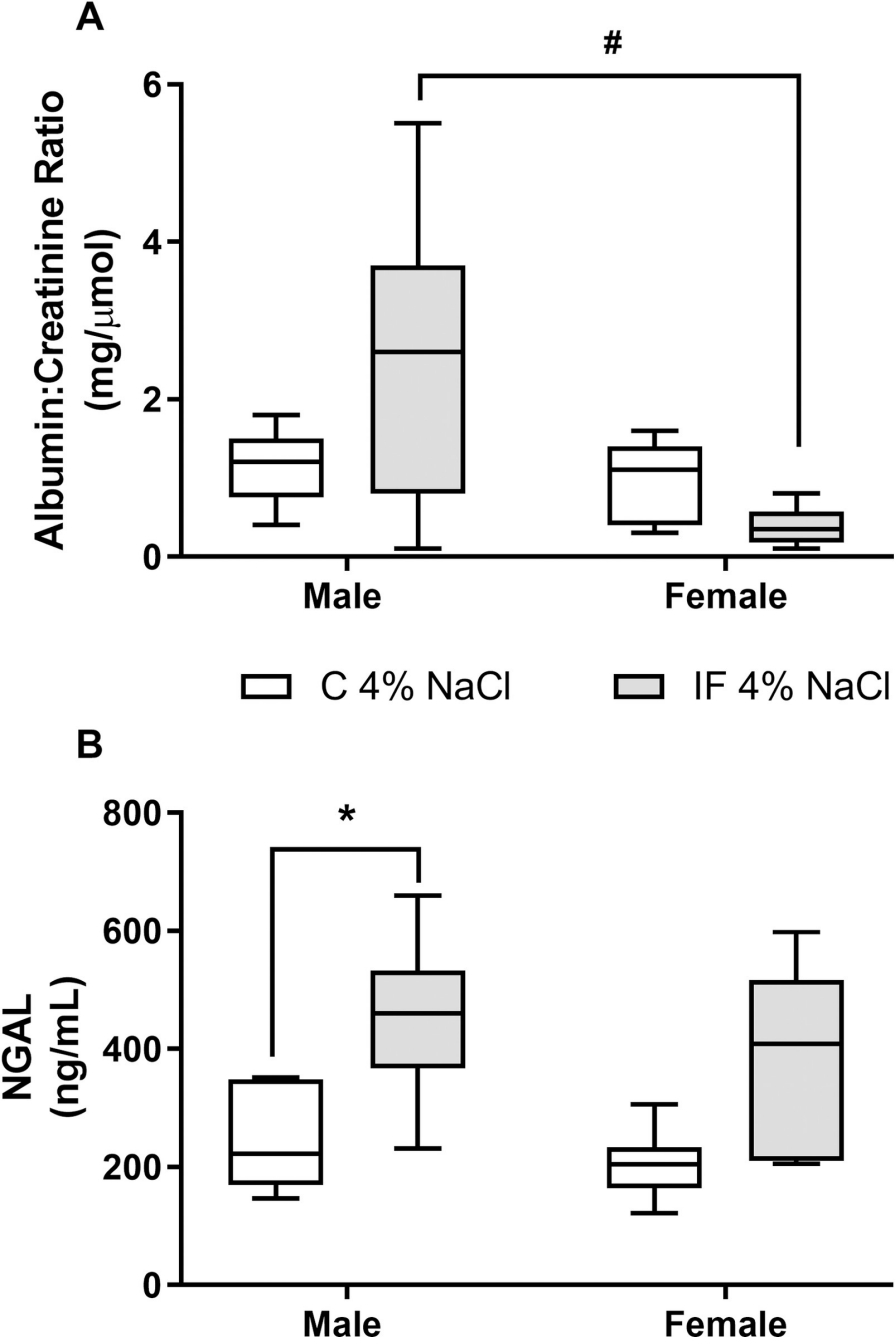
Urinary albumin:creatinine concentration ratio (A) and urinary NGAL concentration (B) in control and IF offspring fed a 4% salt diet from weaning until 12 weeks of age. Male and female control (N = 5 open boxes) and IF (N = 6 shaded boxes) rats were held individually in metabolism cages until they had voided sufficient urine for analysis. Data are presented as box (with median) and whisker plots (5^th^ and 95^th^ centiles). Statistical comparisons were by two-way ANOVA and Tukey’s test. * *P* < 0.05 IF vs control; ^#^ *P* < 0.05 male vs female.

After 8 weeks of salt loading, at 12 weeks of age, spot urine samples were collected to look for markers of renal damage. The urinary albumin:creatinine concentration ratio was significantly higher (*P* < 0.05) in IF males compared to IF females (Fig 6A). Male IF rats also showed significantly elevated (*P* < 0.05) concentrations of NGAL, an early marker of renal injury [20], compared with control males (Fig 6B). IF females tended to have higher urinary NGAL concentrations compared with control females; however this did not reach statistical significance (*P* = 0.066).

**Fig 7:**
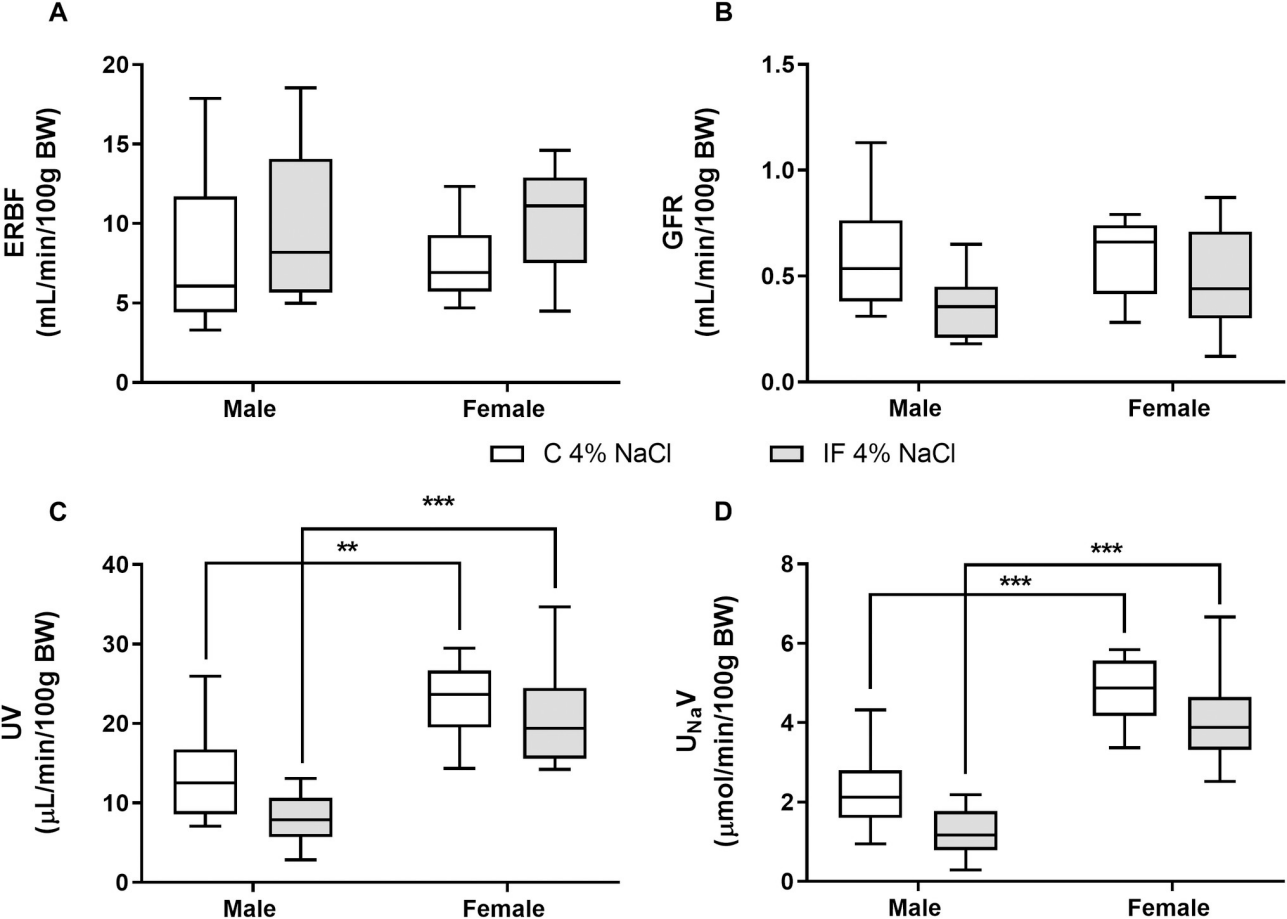
Effective renal blood flow (A), glomerular filtration rate (B), urine flow rate (C) and sodium excretion rate (D) in anaesthetised control and IF offspring fed a 4% salt diet from weaning until 14 weeks of age. Renal haemodynamics (A-B) and urinary excretion rates (C-D) were measured over 3 h during continuous infusion of 0.9% saline at 50 μL/min in male and female control (N = 5 open boxes) and IF (N = 6 shaded boxes) offspring. Data are presented as box (with median) and whisker plots (5^th^ and 95^th^ centiles). Statistical comparisons were by two-way ANOVA and Tukey’s test. ** *P* < 0.01, *** *P* < 0.001 male vs female.

Despite these increases in biomarkers of renal injury, there were no overt changes in renal function when clearance measurements were made at 14 weeks of age. Mean arterial pressure, plasma composition and haematocrit did not differ between IF and control rats maintained on a high salt diet, nor were there any differences between the sexes (Table 7). ERBF and GFR were comparable between IF and controls, as well as across the sexes (Fig 7A and 7B), and UV and U_Na_V remained elevated in females compared with males (*P* < 0.01, Fig 7C and 7D). Similarly, urinary potassium excretion and osmolar excretion were increased significantly (*P* < 0.01) in females compared with males (S6A and S6C Fig), while fractional excretion of potassium and free water clearance were unaltered (S6B and S6D Fig). The only notable difference in renal function in rats maintained on a high salt diet that was not observed in animals fed a standard diet was that the fractional excretion of sodium by IF males (N = 6, 3.3 ± 0.7%) was significantly lower than that of IF females (N = 6, 6.1 ± 0.5%, *P* < 0.05).

**Table 7:** Mean arterial pressure (MAP), plasma electrolyte concentrations, osmolality, protein concentration and haematocrit in anaesthetised control and IF offspring fed at 4% NaCl diet during renal clearance measurements at 14 weeks of age.

	Control (N = 5)		IF (N = 7)	
	Male	Female	Male	Female
MAP (mmHg)	125 ± 4	121 ± 4	116 ± 4	118 ± 3
Na^+^ (mmol/L)	146 ± 4	147 ± 3	142 ± 6	146 ± 2
K^+^ (mmol/L)	3.4 ± 0.2	2.9 ± 0.2	3.5 ± 0.2	3.2 ± 0.1
Osmolality (mOsm/kg H_2_O)	328 ± 16	335 ± 13	334 ± 15	327 ± 11
Protein (g/100 mL)	4.3 ± 0.2	4.4 ± 0.2	3.8 ± 0.2	3.9 ± 0.2
Haematocrit (%)	57.1 ± 1.2	50.4 ± 1.1	56.5 ± 2.6	51.4 ± 2.2

Data are shown as mean ± SEM. Values represent the average per litter; N is the number of litters. Statistical comparisons were by two-way ANOVA or Kruskal-Wallis test. No significant differences were identified.

## Discussion

Having shown previously that exposure to IF during pregnancy resulted in a number of changes in maternal, fetal and placental function [13], the primary aim of the current study was to establish whether the intrauterine challenge posed by IF leads to altered cardiovascular, metabolic or renal function in the offspring. The picture that has emerged is that, in contrast to other models of dietary manipulation during pregnancy in the rat such as the maternal low protein (LP) model [14, 17, 21], IF appeared to have minimal impact on the offspring under basal conditions. It was not until IF offspring were subject to a second, postnatal dietary challenge in the form of salt loading that a susceptibility to renal injury was revealed in males.

We have reported previously that the ~30% reduction in food intake by pregnant rats subjected to the IF regimen was associated with a significant reduction in fetal weight at GD21 [13]. In the current study we allowed rats to deliver at term (23 days) and observed that the birth weight of IF pups was not different from that of controls, even though we consistently found that IF dams delivered ~0.5 day early. The rat fetus normally undergoes rapid growth over the final 2 days of gestation [22, 23]; nonetheless the magnitude of the increase in body weight by IF fetuses is striking, bearing in mind that both fetal sexes exhibit FGR at GD21 [13]. While control fetuses increased their body weight by 79 ± 6% between GD21 and term at 23 days, IF fetuses gained 97 ± 5% in order to catch up and achieve a comparable weight to control pups at birth. This pattern of growth restriction followed by rapid catch up growth contrasts with other models of developmental programming, such as the maternal LP model in which fetal weight was greater at GD21 yet birth weight was reduced compared with controls [24]. In humans, we reported recently in a meta-analysis of the impact of Ramadan fasting during pregnancy that birth weight was unaffected by maternal fasting [2]. Therefore, while the pattern of fetal growth in our IF rat model differs from other developmental programming models, it appears to reflect that seen in human Ramadan fasting as evidenced by the lack of impact on birth weight.

In early postnatal life, growth of IF pups matched that of controls; however from PD18 until weaning at 4 weeks IF pups grew more slowly. Interestingly, rat pups begin to move away from a diet comprising milk alone to one which includes solid food from around PD16-18 [25], implying that the retarded growth of IF pups prior to weaning was not due to a reduction in the quality or quantity of milk provided by the dam. Indeed, the growth retardation continued beyond weaning until week 12. However, this was only apparent in male offspring; IF females grew at the same rate as their control counterparts, indicating that there may be sex-dependent differences in nutrient and energy utilisation which are affected by the *in utero* nutrient environment. In male offspring the mechanism may involve changes in mitochondrial function: a reduction in muscle mitochondrial DNA (mtDNA) has been observed in male rats exposed to a LP diet *in utero* [26]. In contrast mitochondrial function was increased male protein-restricted mice, leading to greater oxidative capacity of muscle, increased energy expenditure and diminished weight gain [27]. A different picture has been reported in female offspring exposed to maternal undernutrition, where postnatal weight gain was associated with leptin resistance [28]. Hence maternal dietary stress appears to affect different parts of the energy regulation pathway in males and females.

Despite the pups having comparable body weights to controls at birth, the growth of key organs was not proportionate in new-born IF pups. The brain:liver weight ratio was reduced in IF pups, suggesting that brain growth had not been spared relative to visceral organ growth. This contrasts with other models of food restriction during pregnancy in which growth of the brain is spared at the expense of visceral organs such as the liver [29]. There are no published data on the impact of maternal fasting during pregnancy on brain growth in humans. However, neonatal head circumference as a proxy of brain growth did not show any difference between infants of fasted and non-fasted mothers [30, 31]. Yet, it is interesting to note that a large census-based study of Muslim populations in Uganda (n = 80,000) and Iraq (n = 250,000) has shown that exposure to Ramadan fasting during the first month of pregnancy increased the offspring’s risk of ‘mental or learning disability’ by 50% and of ‘psychological disability’ by 63% [7]. These surveys were not designed to identify the nature of any cognitive deficit associated with maternal IF. However, nutritional challenges during pregnancy leading to low birth weight [32] and slow postnatal growth [33] have been linked to impaired cognitive function in humans in later life. It is interesting to note, therefore, that Muslim children living in England who were exposed to Ramadan fasting *in utero* during the first trimester in particular achieved significantly lower scores in the Key Stage 1 maths, reading and writing tests (taken in primary schools at the age of 7 years) than either Muslim children who were not exposed to Ramadan fasting *in utero* or to Caribbean children matched for socioeconomic status [34].

Kidney weight was also reduced in IF pups at birth, suggesting that renal development was compromised. Despite this, nephron number was not different from that in controls either at birth or later at PD12. The kidney is particularly vulnerable to nutritional insults during pregnancy. Renal mass and nephron number have been reported to be lower in rat offspring exposed to calorific restriction [35] and LP diets [17] during pregnancy. Nephron deficit in particular has been linked to the development of high blood pressure [36], although there is evidence to suggest that low nephron number and hypertension may be independent features of the LP rat model [37]. We acknowledge that we did not use the gold standard method to determine nephron number in the form of non-biased stereology; nonetheless we did not see a difference between control and IF offspring either at birth when nephrogenesis is still underway or at PD12 when nephrogenesis is complete in the rat [19]. The reason for the apparent mismatch between reduced kidney weight at birth and unaltered nephron number in IF offspring is unclear. Li *et al*. [38] have reported that pre-term babies exhibit disproportionate growth of the renal compartments: the cortex had undergone hypertrophy whereas the medulla was under developed compared with term babies at 6 months of age. This raises the possibility that despite the apparent lack of a nephron deficit in IF offspring, growth rates across the renal compartments may have differed between IF and control kidneys. Further studies are necessary to confirm this notion. It is also of interest to note that, in contrast to the food-only model of IF described in the current study, a 30% reduction in nephron number has observed in rat offspring when pregnant dams were subjected to intermittent restriction of both food and water for the whole of pregnancy [39]; however shorter periods of food and water restriction (3 days which is equivalent to 1 month of human pregnancy) had no effect on nephron count [40].

Blood pressure, first measured at 5 weeks and finally at 14 weeks at the end of the study, did not differ between IF and control offspring of either sex. This is in marked contrast to other models of developmental programming, including calorific restriction [41], high fat [42] and LP diets [14] as well as fetal exposure to glucocorticoids [43], in which hypertension is a common feature. It is possible that IF rats may go on to develop high blood pressure later in life: Kahn *et al*. [42] reported that the female offspring of dams fed a high fat diet were not hypertensive until 180 days old, and that males still had not developed high blood pressure at 360 days of age. Conversely, we have reported previously that offspring exposed to a LP diet have elevated blood pressure from as early as 4 weeks of age [17]. As there is variability in the timescales over which hypertension can develop in rat models of developmental programming, we would need to assess blood pressure in IF rat offspring over a longer period before we could confirm that the offspring do not develop hypertension later in life.

Renal function did not differ between adult (14 week old) IF and control offspring under basal conditions, which is in accord with the absence of any change in either blood pressure or extracellular fluid volume. We have reported previously that renal haemodynamics, assessed in the same manner as the current study, are unaltered in 4 week old LP rats [17], while others have recorded a reduction in creatinine clearance (a marker of glomerular filtration rate) at this age [16]. Glomerular filtration rate did not differ in older LP rats (20 weeks); however albuminuria was present [16] indicating that the glomerular filtration barrier was damaged. Renal tubular function in the LP rat does, however, appear to be impaired from an early age. We observed a natriuresis and diuresis in LP rats aged 4 weeks, which appeared to be driven by a reduction in Na^+^K^+^ATPase activity in the renal medulla [44]. Despite the loss of sodium, LP rats had increased extracellular fluid volume [18] and raised blood pressure [44]. Hence it appears that renal function and extracellular fluid volume regulation in the LP rat differs from that in the IF model.

In an attempt to uncover any underlying deficit in kidney function, we challenged a separate group of IF rats by weaning them onto a high (4%) salt diet prior to assessing their renal function at 14 weeks of age. Salt loading revealed subtle, but important, differences between IF and control animals. For example, the high salt diet resulted in comparable increases in blood pressure in both IF and control animals. However, blood pressure in the IF rats began to increase at an earlier age than their control counterparts. This was particularly apparent in the IF males which became hypertensive at 7 weeks whereas blood pressure did not begin to increase in control males until 10 weeks of age. Renal haemodynamics did not differ between IF and control rats maintained on a high salt diet and although the urinary sodium excretion rate was higher than that in animals maintained on a standard (1%) salt diet, it did not differ between IF and control animals fed a high salt diet. Yet there were indications that the high salt diet had affected the kidneys of male IF rats. The urinary albumin:creatinine concentration ratio, a marker of glomerular damage [45], and urinary NGAL concentration, an early marker of renal injury [20], were both elevated in IF males. NGAL is produced by injured renal tubules and as a result of macrophage infiltration following inflammation [46], which is commonly associated with dietary salt loading [47]. The marked increase in urinary NGAL concentration in IF males therefore suggests that they are more susceptible to renal inflammation following salt loading compared with their control counterparts. This in turn raises the possibility that IF males, in particular, may be more prone to a deterioration in renal function later in life.

The only other differences in renal function that we observed in rats fed either a 1% or 4% salt diet was between the sexes. This reflects, in part, the experimental design as infusion rates were not adjusted for body weight. Rather, in common with many other studies of renal function [48, 49] as well as previous reports from our laboratory [17, 44], urinary outputs were adjusted for body weight instead. We acknowledge that this is a limitation of our study; nonetheless if there had been sex-related differences in the IF animals the sex * diet interaction term in the ANOVA would have been able to highlight such effects. Therefore, it is unlikely that the approach taken obscured any sex-related effect of exposure to IF.

In addition to assessing the impact of salt loading on blood pressure and renal function, we also determined the salt preference and aversion threshold of IF rats. When given a choice between water and low concentration solutions of saline to drink, rats express a preference for saline [50]. This salt preference is increased in several models of developmental programming, including the LP rat [18], Dahl salt-sensitive rats exposed to a low sodium diet *in utero* [51] and rats whose mothers underwent a partial aortic ligation [52]. Similarly maternal dehydration, which can be induced in the rat by subcutaneous injection of polyethylene glycol in order to mimic human vomiting during pregnancy, also increased salt preference in the offspring [53]. It is interesting to note in this context that the risk of hyperemesis gravidarum (HG—morning sickness) is increased among pregnant women fasting during Ramadan, particularly in the first trimester [54], suggesting perhaps that their offspring may have a greater preference for salt. HG itself is associated with higher blood pressure in children age 5–6 years [55] which is coupled with lower insulin sensitivity and elevated cortisol concentrations [56] and greater risk of neuropsychiatric disorders including depression, bipolar disorder and anxiety [57]. Therefore HG must be considered as a potential confounder when interpreting the outcomes of Ramadan fasting studies in humans.

In contrast to other developmental programming models, no such enhanced preference for salt was observed in the IF rat model. Both male and female IF rats drank similar quantities of saline, as a proportion of their total fluid intake, compared with their control counterparts. There was, however, a strong sex difference. Both IF and control females drank more fluid relative to their body weight compared with males, and of that fluid, the females drank significantly more saline (72–79% of total fluid intake) compared with their male counterparts (51–54%). Similar sex-dependent differences in salt preference have been noted before and have been attributed to oestrogen-mediated blunting of salt-sensitivity in female rats [58].

Although there was no within-sex difference in the salt preference of IF and control rats, there was a difference in the salt aversion threshold of IF females compared with control females. Control females began to show an aversion to saline when the concentration reached 1.5% whereas the IF females tolerated concentrations up to 1.8%. The literature in this area is unclear: in males the point of indifference (the concentration of saline at which rats express equal preference for water and saline) is reported to be at 1.5% in both neonatal and adult rats; saline solutions of 3% or higher are rejected completely [59]. Female rats are able to detect lower concentrations of saline than males, while also being more tolerant of higher concentrations [58]. However, the aversion threshold for saline does not appear to have been reported in female rats. Oestrogen has been shown to regulate salt-sensitivity in female rats [58]; however we have no data on sex steroids or their receptors in IF rats upon which to base a hypothesis to explain the observed difference in the salt aversion threshold.

Both male and female IF offspring responded to glucose and insulin challenges in the same manner as control rats, indicating that they were not insulin-resistant at 12 weeks of age. Impaired glucose tolerance and insulin resistance have been reported in other models of developmental programming, including calorific restriction [60] and LP diets [15], but again age seems to be critical. Hales *et al*. [61] reported that LP offspring were actually more glucose tolerant than controls at 3 months of age; it was not until they were 15 months old that they became glucose intolerant and exhibited frank diabetes [15]. MicroRNA (miRNA) may act as an early marker of the risk of developing diabetes in later life. Ferland-McCollough *et al*. [62] reported that expression of miRNA-483-3p, which regulates growth/differentiation factor 3 (GDF3), is upregulated as early as 22 days of age in LP rats and remained increased at 3 months of age. These authors proposed that, as a result of increased expression of miRNA-483-3p, GDF3 is downregulated which in turn affects the ability of adipose cells to store lipids and ultimately leads to insulin resistance. It would therefore be of interest to assess miRNA-483-3p expression in IF rats, as a potential early marker of insulin resistance and diabetes in later life.

A limitation of this study is that the pregnant dams were fasted for the whole of pregnancy. Ramadan takes place over a lunar month, which is approximately equivalent to 3 days of a rat pregnancy. Ramadan could fall at any stage of a woman’s pregnancy, so in order to assess the effects of exposure to IF at all stages of fetal development and to maximise the potential impact upon the offspring we elected to fast animals for the whole of pregnancy. This approach has the advantage that it captures the potential impact of IF on the development of multiple organ systems in the same animal, thereby reducing the overall number of animals necessary in accordance with the 3Rs. However, we acknowledge that it does not fully replicate the pattern of fasting as practiced by Muslims; therefore in future studies a more targeted approach will be taken, focussing on critical periods of development e.g. early pregnancy for development of the brain or GD13-15 for development of the metanephros.

In conclusion, this study has shown that exposure to IF *in utero* appears to have minimal impact on the cardiovascular, metabolic and renal health of adult offspring, contrasting with offspring outcomes from other developmental programming models in which maternal dietary intake is altered during pregnancy. Despite experiencing FGR as a result of altered placental nutrient transport function [13], IF pups have comparable birth weights with controls. As they grow, blood pressure remains normal, glucose and insulin tolerance are unaltered and basal renal function is unaffected up to the age of 14 weeks. When challenged by a dietary salt load, blood pressure begins to increase sooner in IF rats compared with controls; however the magnitude of the overall hypertensive response is no different. The kidneys of IF rats appear to be able to accommodate the increase in sodium intake; however there are indications that they have undergone injury as male IF rats in particular exhibited albuminuria and had raised urinary concentrations of the kidney injury marker, NGAL. Therefore, IF rats may be more susceptible to renal injury.

## Supporting information

S1 Fig Study design (A) normal 1% salt diet and (B) high 4% salt diet. Food was removed from IF rats for 16 h per day between 17:00 and 09:00 from GD1 until GD22; water was available *ad libitum*. Control rats had free access to food and water at all times. (A) The offspring from N = 16 control and N = 19 IF dams were weaned onto standard chow containing 1% NaCl at 4 weeks of age. Systolic blood pressure (SBP) was measured in all offspring at 5, 7 and 10 weeks. Offspring were randomly allocated to nephron counting (postnatal (PD) days 1 and 12; glucose tolerance test (GTT) or insulin tolerance test (ITT) at week 12 or measurement of renal function at 14 weeks. (B) The offspring from N = 5 control and N = 6 IF dams were weaned onto standard chow containing 1% NaCl or chow containing 4% NaCl at 4 weeks of age. SBP was measured in all offspring at 5, 7 and 10 weeks. Offspring were randomly allocated to saline preference testing at week 7, measurement of extracellular fluid volume at week 12 or measurement of renal function at 14 weeks. N = number of dams, n = number of offspring; where fewer than the planned number of offspring were included in the final data set (e.g. due to technical failures) the actual n number is indicated in the relevant legend.
(TIF)

S2 Fig Maternal food intake (A), water intake (B) and weight gain (C) in pregnant rats throughout gestation. Food was removed from IF rats (N = 13 open squares) for 16 h per day between 17:00 and 09:00 from GD1 until GD22; water was available *ad libitum*. Control rats (N = 11 closed squares) had free access to food and water at all times. Data are presented as mean ± SEM, except when SEM falls within the size of the symbol. Statistical comparisons were by two-way ANOVA with repeated measures and Tukey’s test. * *P* < 0.05, ** *P* < 0.01, *** *P* < 0.001 IF vs control.
(TIF)

S3 Fig Potassium excretion rate (A), fractional excretion of potassium (B), osmolar excretion rate (C) and free water clearance (D) in anaesthetised control and IF offspring at 14 weeks of age. Urinary excretion was measured over 3 h during continuous infusion of 0.9% saline at 50 μL/min in male and female control (N = 5 open boxes) and IF (N = 7 shaded boxes) offspring. Data are presented as box (with median) and whisker plots (5^th^ and 95^th^ centiles). Statistical comparisons were by two-way ANOVA and Tukey’s test. ** *P* < 0.01, *** *P* < 0.001 male vs female.
(TIF)

S4 Fig Salt aversion threshold in control (A, C) and IF (B, D) offspring. Male (A, B) and female (C, D) control (N = 5) and IF (N = 6) rats were offered a choice of water or saline increasing in concentration from 0.9% to 2.1% as drinking fluid. Saline intake is shown as a percentage of total fluid intake over 3 consecutive days for each concentration. Data are presented as mean + SEM. Statistical comparisons were by one-way ANOVA and Dunnett’s test. * *P* < 0.05, ** *P* < 0.01, *** *P* < 0.001 vs 0.9% saline.
(TIF)

S5 Fig Food intake (A, B) and water intake (C, D) of control and IF offspring fed either a 1% or 4% salt diet from 4 weeks to 12 weeks of age. Male (circles) and female (squares) control (N = 5 per diet A, C) and IF (N = 5 per diet B, D) rats were weaned at 4 weeks of age onto a diet containing either 1% NaCl (NS–normal salt, closed symbols) or 4% NaCl (HS–high salt, open symbols). Data are presented as mean ± SEM, except when SEM falls within the size of the symbol. Statistical comparisons were by two-way ANOVA with repeated measures and Tukey’s test. * *P* < 0.05, *** *P* < 0.001 HS diet vs NS diet.
(TIF)

S6 Fig Potassium excretion rate (A), fractional excretion of potassium (B), osmolar excretion rate (C) and free water clearance (D) in anaesthetised control and IF offspring fed a 4% salt diet from weaning until 14 weeks of age. Urinary excretion was measured over 3 h during continuous infusion of 0.9% saline at 50 μL/min in male and female control (N = 5 open boxes) and IF (N = 6 shaded boxes) offspring. Data are presented as box (with median) and whisker plots (5^th^ and 95^th^ centiles). Statistical comparisons were by two-way ANOVA and Tukey’s test. * *P* < 0.05 IF vs control; ^##^ *P* < 0.01, ^###^ *P* < 0.001 male vs female.
(TIF)

S1 Dataset (XLSX)
