# Supplementary figures and images for: Impact of maternal intermittent fasting during pregnancy on cardiovascular, metabolic and renal function in adult rat offspring
Source: PLoS One. 2022 Mar 10;17(3):e0258372. doi: 10.1371/journal.pone.0258372 (PMC8912128; doi:10.1371/journal.pone.0258372)

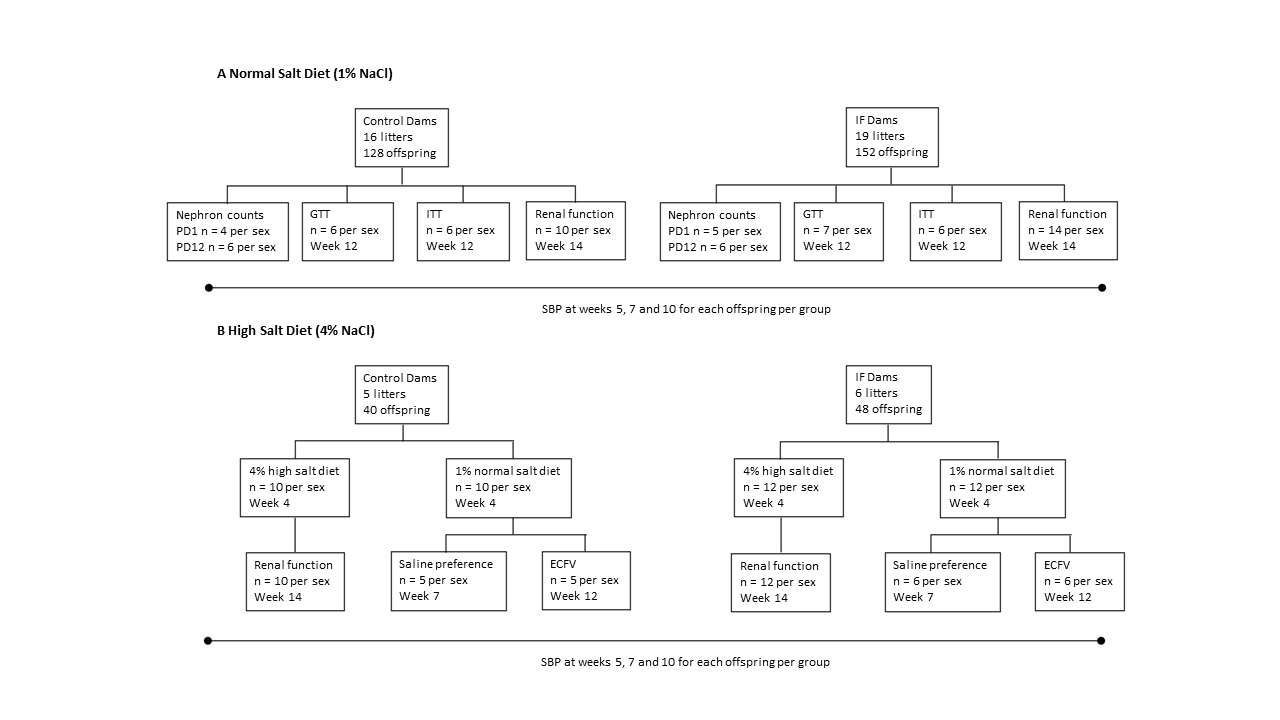

Supplement: S1 Fig — Study design (A) normal 1% salt diet and (B) high 4% salt diet. Food was removed from IF rats for 16 h per day between 17:00 and 09:00 from GD1 until GD22; water was available ad libitum. Control rats had free access to food and water at all times. (A) The offspring from N = 16 control and N = 19 IF dams were weaned onto standard chow containing 1% NaCl at 4 weeks of age. Systolic blood pressure (SBP) was measured in all offspring at 5, 7 and 10 weeks. Offspring were randomly allocated to nephron counting (postnatal (PD) days 1 and 12; glucose tolerance test (GTT) or insulin tolerance test (ITT) at week 12 or measurement of renal function at 14 weeks. (B) The offspring from N = 5 control and N = 6 IF dams were weaned onto standard chow containing 1% NaCl or chow containing 4% NaCl at 4 weeks of age. SBP was measured in all offspring at 5, 7 and 10 weeks. Offspring were randomly allocated to saline preference testing at week 7, measurement of extracellular fluid volume at week 12 or measurement of renal function at 14 weeks. N = number of dams, n = number of offspring; where fewer than the planned number of offspring were included in the final data set (e.g. due to technical failures) the actual n number is indicated in the relevant legend. (TIF) [file pone.0258372.s001.tif]

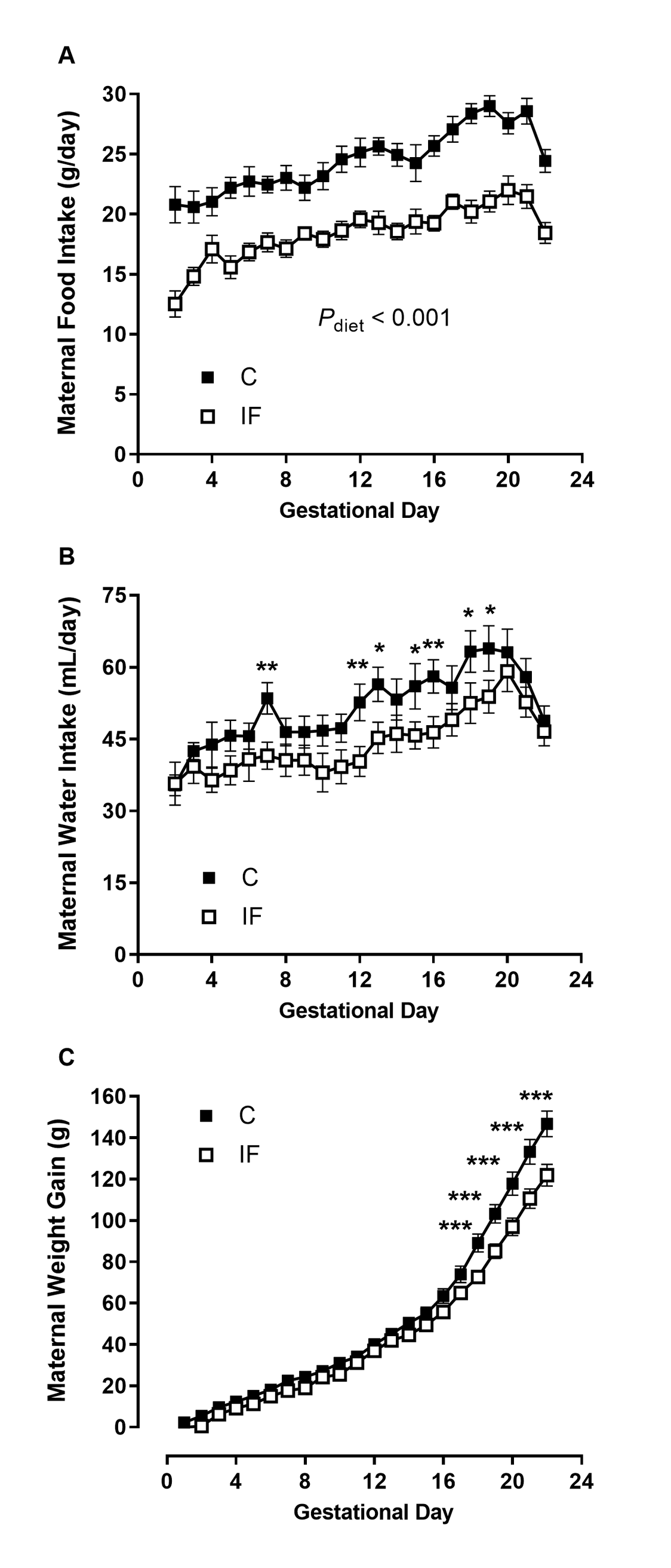

Supplement: S2 Fig — Maternal food intake (A), water intake (B) and weight gain (C) in pregnant rats throughout gestation. Food was removed from IF rats (N = 13 open squares) for 16 h per day between 17:00 and 09:00 from GD1 until GD22; water was available ad libitum. Control rats (N = 11 closed squares) had free access to food and water at all times. Data are presented as mean ± SEM, except when SEM falls within the size of the symbol. Statistical comparisons were by two-way ANOVA with repeated measures and Tukey’s test. * P < 0.05, ** P < 0.01, *** P < 0.001 IF vs control. (TIF) [file pone.0258372.s002.tif]

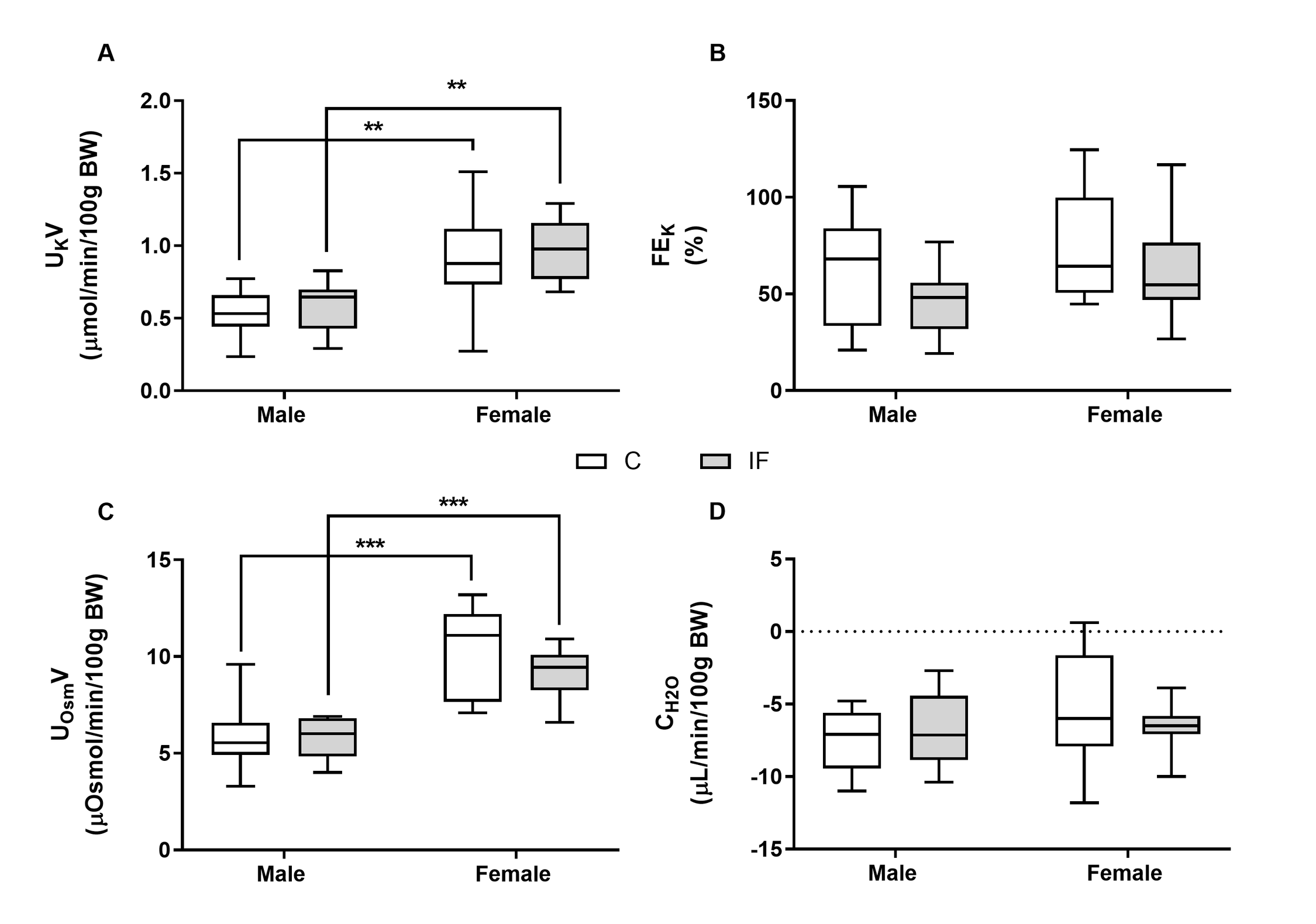

Supplement: S3 Fig — Potassium excretion rate (A), fractional excretion of potassium (B), osmolar excretion rate (C) and free water clearance (D) in anaesthetised control and IF offspring at 14 weeks of age. Urinary excretion was measured over 3 h during continuous infusion of 0.9% saline at 50 μL/min in male and female control (N = 5 open boxes) and IF (N = 7 shaded boxes) offspring. Data are presented as box (with median) and whisker plots (5th and 95th centiles). Statistical comparisons were by two-way ANOVA and Tukey’s test. ** P < 0.01, *** P < 0.001 male vs female. (TIF) [file pone.0258372.s003.tif]

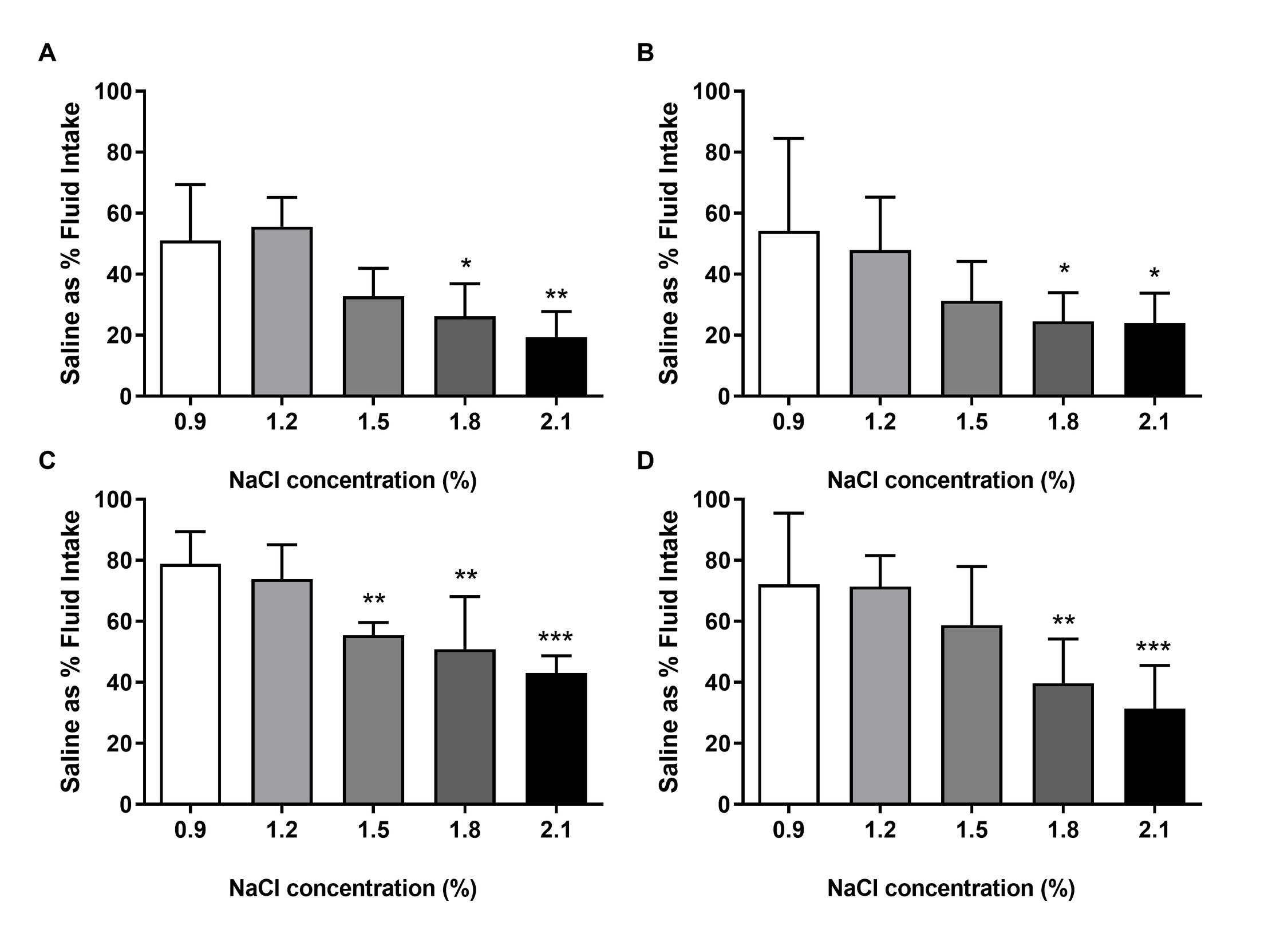

Supplement: S4 Fig — Salt aversion threshold in control (A, C) and IF (B, D) offspring. Male (A, B) and female (C, D) control (N = 5) and IF (N = 6) rats were offered a choice of water or saline increasing in concentration from 0.9% to 2.1% as drinking fluid. Saline intake is shown as a percentage of total fluid intake over 3 consecutive days for each concentration. Data are presented as mean + SEM. Statistical comparisons were by one-way ANOVA and Dunnett’s test. * P < 0.05, ** P < 0.01, *** P < 0.001 vs 0.9% saline. (TIF) [file pone.0258372.s004.tif]

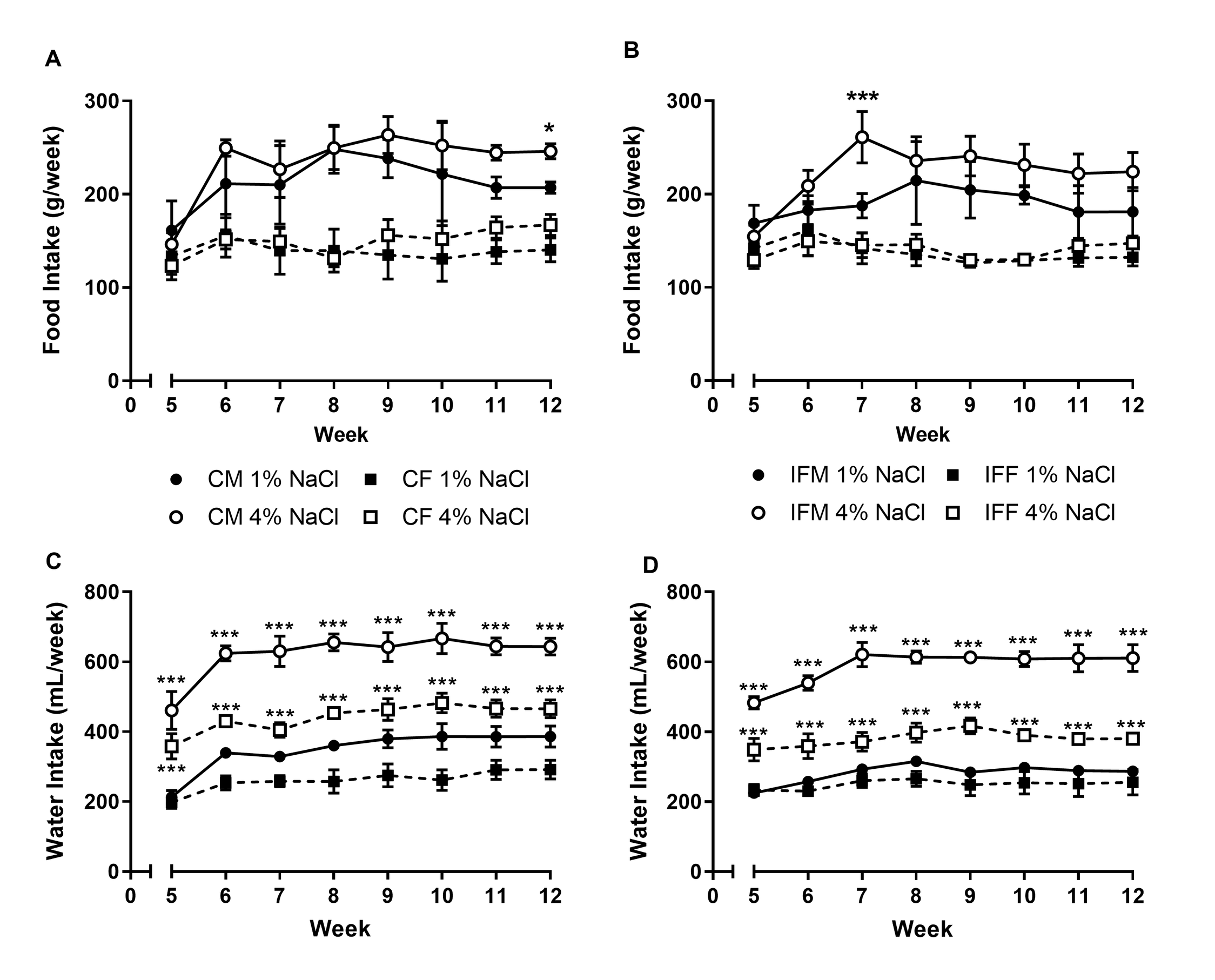

Supplement: S5 Fig — Food intake (A, B) and water intake (C, D) of control and IF offspring fed either a 1% or 4% salt diet from 4 weeks to 12 weeks of age. Male (circles) and female (squares) control (N = 5 per diet A, C) and IF (N = 5 per diet B, D) rats were weaned at 4 weeks of age onto a diet containing either 1% NaCl (NS–normal salt, closed symbols) or 4% NaCl (HS–high salt, open symbols). Data are presented as mean ± SEM, except when SEM falls within the size of the symbol. Statistical comparisons were by two-way ANOVA with repeated measures and Tukey’s test. * P < 0.05, *** P < 0.001 HS diet vs NS diet. (TIF) [file pone.0258372.s005.tif]

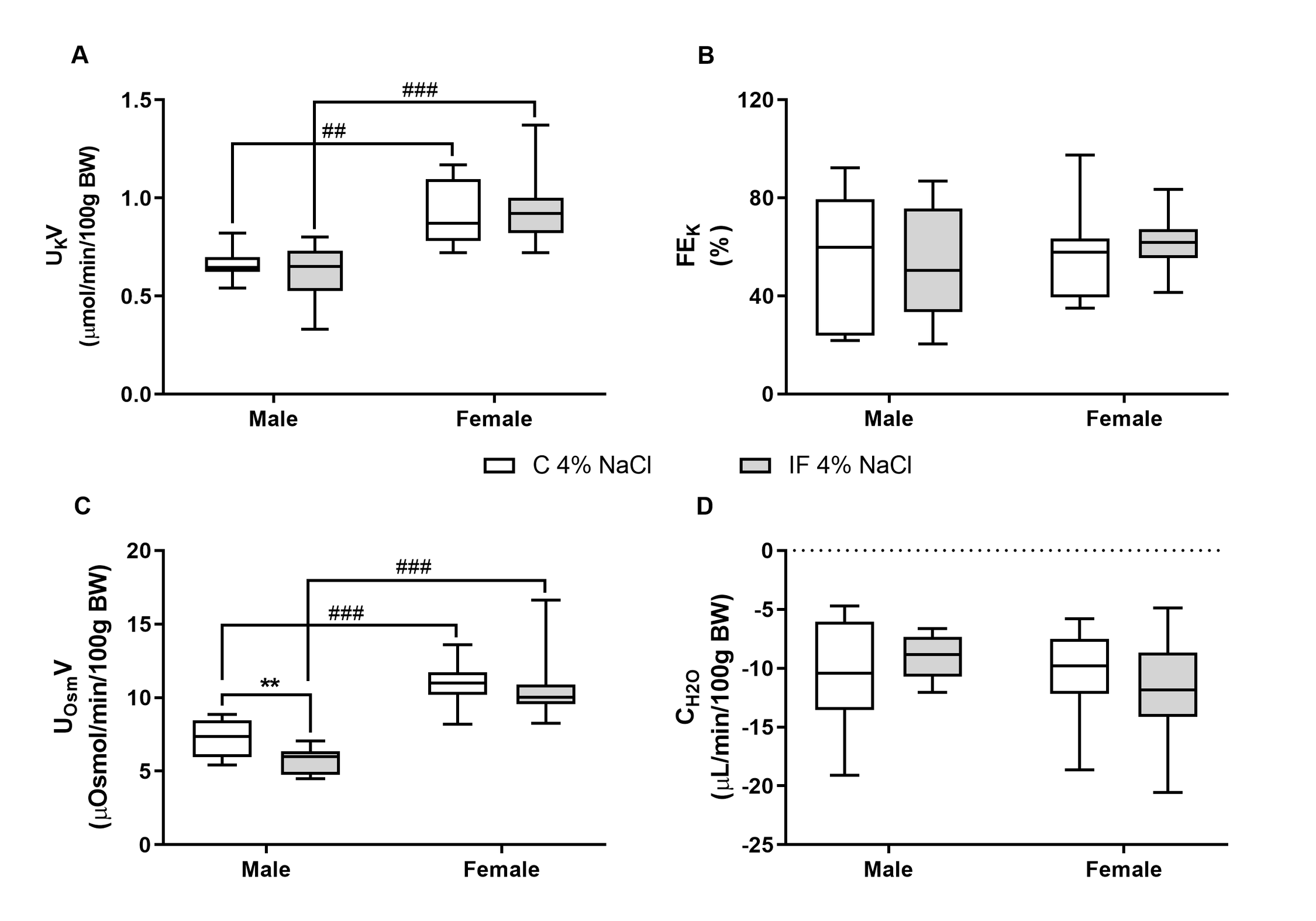

Supplement: S6 Fig — Potassium excretion rate (A), fractional excretion of potassium (B), osmolar excretion rate (C) and free water clearance (D) in anaesthetised control and IF offspring fed a 4% salt diet from weaning until 14 weeks of age. Urinary excretion was measured over 3 h during continuous infusion of 0.9% saline at 50 μL/min in male and female control (N = 5 open boxes) and IF (N = 6 shaded boxes) offspring. Data are presented as box (with median) and whisker plots (5th and 95th centiles). Statistical comparisons were by two-way ANOVA and Tukey’s test. * P < 0.05 IF vs control; ## P < 0.01, ### P < 0.001 male vs female. (TIF) [file pone.0258372.s006.tif]
